# A tale of two serines: the effects of histone H2A mutations S122A and S129A on chromosome nondisjunction in *Saccharomyces cerevisiae*

**DOI:** 10.1093/genetics/iyae194

**Published:** 2024-11-18

**Authors:** Stanislav G Kozmin, Margaret Dominska, Robert J Kokoska, Thomas D Petes

**Affiliations:** Department of Molecular Genetics and Microbiology, Duke University School of Medicine, Duke University, Durham, NC 27710, USA; Department of Molecular Genetics and Microbiology, Duke University School of Medicine, Duke University, Durham, NC 27710, USA; Biological and Biotechnology Sciences, DEVCOM-ARL Army Research Office, 800 Park Offices Drive, Durham, NC 27703, USA; Department of Molecular Genetics and Microbiology, Duke University School of Medicine, Duke University, Durham, NC 27710, USA

**Keywords:** chromosome nondisjunction, histone mutations, *bub1*, *tel1*, *mec1*, uniparental disomy

## Abstract

Near the C-terminus of histone H2A in the yeast *Saccharomyces cerevisiae*, there are 2 serines (S122 and S129) that are targets of phosphorylation. The phosphorylation of serine 129 in response to DNA damage is dependent on the Tel1 and Mec1 kinases. In *Schizosaccharomyces pombe* and *S. cerevisiae*, the phosphorylation of serine 122 is dependent on the Bub1 kinase, and *S. pombe* strains with an alanine mutation of this serine have elevated levels of lagging chromosomes in mitosis. Strains that lack both Tel1 and Mec1 in *S. cerevisiae* have very elevated rates of nondisjunction. To clarify the functional importance of phosphorylation of serines 122 and 129 in H2A, we measured chromosome loss rates in single-mutant strains and double-mutant combinations. We also examined the interaction of mutations of *BUB1*, *TEL1*, and *MEC1* in combination with mutations of serines 122 and 129 in H2A. We conclude that the phosphorylation state of S129 has no effect on chromosome disjunction whereas mutations that inactivate Bub1 or a S122A mutation in the histone H2A greatly elevate the rate of chromosome nondisjunction. Based on this analysis, we suggest that Bub1 exerts its primary effect on chromosome disjunction by phosphorylating S122 of histone H2A. However, Tel1, Mec1, and Bub1 are also functionally redundant in a second pathway affecting chromosome disjunction that is at least partially independent of phosphorylation of S122 of H2A.

## Introduction

Many mutations in the yeast *Saccharomyces cerevisiae* result in elevated levels of aneuploidy (extra or missing chromosomes). Although aneuploidy can be advantageous under some types of environmental stress (Gilchrist and Stelkens 2019), most aneuploid strains have partial growth defects (Torres *et al*. 2007). Aneuploidy can be caused by a variety of mechanisms (for example, failure to repair endogenous DNA damage, loss of chromosome cohesion, and others), but 1 common class is mutants affecting the function of the kinetochore (Stirling *et al*. 2012). As described below, mutations of the histone H2A sometimes lead to high levels of chromosome nondisjunction (Pinto and Winston 2000).

*S. cerevisiae* strains that have serine to alanine mutations in positions 122 or 129 of H2A are sensitive to DNA damage by methyl methanesulfonate (Downs *et al*. 2000; Harvey *et al*. 2005). Different publications use different numbering systems for the amino acids depending on whether the N-terminal methionine is included; below, we will use the numbering system that includes this methionine. The serine at 129 is adjacent to a glutamine residue, and the SQ motif is one target of PI-3-like kinases (Kim *et al*. 1999) such as Tel1 and Mec1 (Mallory and Petes 2000). Strains that lack the Tel1p and Mec1p kinases fail to phosphorylate S129 in H2A in response to methyl methanesulfonate (Downs *et al*. 2000) or DNA double-stranded breaks (Shroff *et al*. 2004; Unal *et al*. 2004). Kawashima *et al*. (2010) showed that the Bub1 kinase phosphorylates H2A on S122 and that failure to phosphorylate S122 results in loss of shugoshin from the centromere. The Sgo1 and Sgo2 shugoshin proteins have several roles in ensuring accurate chromosome segregation such as protecting sister chromatin cohesion and recruiting other important centromere-binding proteins (Yao and Dai 2012). *Schizosaccharomyes pombe* strains with the S122A mutation of H2A or with the *bub1* mutation have a substantially elevated frequency of lagging chromosomes in mitosis and a high rate of meiotic nondisjunction (Kawashima *et al*. 2010).

In *Candida albicans*, phosphorylation of a serine in H2A located at S122 is important for accurate chromosome segregation (Brimacombe *et al*. 2019). *C. albicans* has 2 H2A genes, *H2A.1* and *H2A.2*. *H2A.1* and *H2A.2* have lysine and serine at position 122, respectively. *C. albicans* strains that lack H2A.2 have a substantially elevated rate of chromosome loss, similar to the levels observed in strains that lack Bub1 or Sgo1.

A number of other yeast mutants have very elevated levels of chromosome nondisjunction. Tel1 and Mec1 are kinases with multiple targets that affect a variety of cellular functions including telomere length regulation and DNA repair. *S. cerevisiae* strains with mutations in both *TEL1* and *MEC1* have very elevated rates of deletions and translocations relative to single-mutant strains, suggesting functional redundancy of Tel1p and Mec1p (Myung *et al*. 2001; Craven *et al*. 2002). Functional redundancy of the 2 kinases is also demonstrated by the level of phosphorylation detected on target proteins. For example, loss of Mec1 substantially reduces the level of phosphorylation of S129A of H2A, whereas strains that lack both Mec1 and Tel1 have no detectable phosphorylation of this residue (Downs *et al*. 2000). Many targets of these enzymes, including proteins involved in the DNA damage response, have been identified; Bub1 is one potential target (Cheung *et al*. 2012).

Yeast strains with the *tel1mec1* genotype have very elevated rates of several types of genome alterations: translocations, large deletions/duplications, and aneuploidy (McCulley and Petes 2010). These phenotypes reflect 2 different effects of the double mutation. A major contributor to the elevated rate of chromosome rearrangements is the telomere defect in *tel1mec1* strains. The short telomeres of *tel1mec1* strains lead to frequent telomere–telomere fusions and subsequent chromosome rearrangements (Mieczkowski *et al*. 2003). Expression of a protein in which telomerase is fused to a telomere-binding protein substantially suppresses the chromosome instability phenotype of *tel1mec1* strains, but has no significant effect on the high levels of aneuploidy (McCulley and Petes 2010).

In this report, we compare the levels of aneuploidy in *S. cerevisiae* strains with mutations in S122A of H2A, S129A of H2A, *bub1*, and *tel1mec1*, and in strains with various combinations of double and triple mutants. We found that strains with the S129A mutation of H2A had very low (wild-type) levels of chromosome nondisjunction; most of the other mutant strains had very elevated levels (100-fold or more) of nondisjunction. Epistasis analysis indicates that both Bub1 and Tel1/Mec1 act in a pathway with H2A as a downstream target. We also suggest the possibility of a minor pathway in which Tel1/Mec1 and Bub1 have redundant activities affecting chromosome segregation independently of H2A.

## Materials and methods

### Strain construction

The genotypes of the strains are in Supplementary File 1 and details of the strain constructions are in Supplementary Materials and Methods. Most of the diploids used in the study were derived from crosses of derivatives of the related *S. cerevisiae* haploid strains FY406 (isogenic with S288c; Hirschhorn *et al*. 1995) and MD761-5-16A. The genotype of FY406 is as follows: *MAT***a***(hta1-htb1)Δ::LEU2 (hta2-htb2)Δ::TRP1ura3-52 leu2Δ1 lys2-128Δ his3Δ200 trp1Δ63 pAB6 (HTA1-HTB1; URA3)*; pAB6 contains the wild-type *HTA1* and *HTB1* genes in the centromere-containing vector pRS316 (Hirschhorn *et al*. 1995). MD761-5-16A has the genotype: *MAT*α *(hta1-htb1)Δ::LEU2 (hta2-htb2)Δ::TRP1leu2 his3 ura3 lys2-128Δ trp1 can1-100 pAB6 (HTA1-HTB1; URA3)*. Most of the diploids in this study had homozygous deletions of the chromosomal copies of the 2 H2A- and H2B-encoding histone genes (*HTA1-HTB1* and *HTA2-HTB2*) with centromere-containing plasmids containing either the wild-type *HTA1* and *HTB1* genes or mutant variants of the *HTA1* gene and the wild-type *HTB1* gene. In addition, these strains were wild-type (control strain) or homozygous of mutations in the *TEL1*, *MEC1*, *BUB1*, and/or *SML1* genes.

### Measurements of the rate of loss of chromosome I

In order to monitor the rate of chromosome loss, we constructed strains heterozygous for a *URA3* insertion located about 170 bp from the centromere of chromosome I (details in Supplementary Materials and Methods). Derivatives of these strains that were 5-fluoroorotic acid (5-FOA) resistant can be generated by loss of chromosome I, mitotic recombination resulting in loss of *URA3*, or a mutation in the wild-type *URA3* gene. By PCR analysis, we confirmed that all of the 5-FOA-resistant derivatives had lost the wild-type *URA3* gene, ruling out mutations of *URA3* in the generation of the 5-FOA^R^ derivatives. We used 2 sets of primers for this analysis: URA3-intF (5′ TTGATGTTAGCAGAATTGTC) and cen1-dd (5′ TACTTCCTGACTCCTTCAAG) producing a fragment of about 1.4 kb and URA3-intR (CTAATGCTTCAACTAACTCC) and cen1-verF (5′ GAGCTTTCATTTCAAGCGCC) producing a fragment of about 5.5 kb.

To calculate a rate of chromosome loss, we measured the frequency of 5-FOA-resistant derivatives in at least 40 independent cultures and converted these frequency measurements into rates by the method of the median fluctuation analysis (Lea and Coulson 1949; Kiktev *et al*. 2018). In addition, in some of the strains (Supplementary File 1), the chromosome I homolog with the *URA3* insertion also had an insertion of *hyg* near the left end of the chromosome. We found that 5-FOA-resistant derivatives of these strains were usually sensitive to hygromycin, as expected for chromosome loss.

In comparing rates of different genotypes that had large 95% confidence limits, we used a second method for calculating significant differences. Individual 5-FOA-resistant frequencies for each genotype were compared with 5-FOA-resistant frequencies of other genotypes in pairwise combinations by the Wilcoxon–Mann–Whitney test using the Prism 10 program (GraphPad Software, USA).

### Analysis of chromosome loss by DNA microarrays

One method that we used to look for aneuploidy was comparative genomic hybridization (CGH) microarrays. Microarrays with yeast genomic sequences were obtained from Agilent Technologies (G4499A, Custom ChIP-on-chip (8 × 15) with configuration: 6 digit Amadid #028943). DNA preparation, hybridization conditions, and microarray analysis were done as described in McCulley and Petes (2010). In brief, DNA from the control euploid strain W303-1A (Matheson *et al*. 2017) was labeled with Cy3 dUTP and DNA from the experimental DNA was labeled with Cy5 dUTP. The samples were mixed and hybridized to the microarray. The microarrays were scanned using a GenePix 4000B scanner (Axon Instruments), and the ratio of hybridization to the 2 probes was measured throughout the genome. The amounts of hybridization were normalized such that the signal for Cy3- and Cy5-labeled samples was equal.

### Genetic methods and media

Standard methods of transformation, mating, and tetrad dissection were used. Most of the media used was also standard (Rose *et al*. 1990). The medium to detect 5-FOA-resistant isolates contained 1 g/L of 5-FOA and 50 mm/L of uracil.

## Results

*S. cerevisiae* strains with mutations in serine 122 of histone H2A (*hta1-S122A*), *bub1*, and *tel1mec1* have very high frequencies of chromosome nondisjunction (Warren *et al*. 2002; Kawashima *et al*. 2010; McCulley and Petes 2010; Uffenbeck 2013). However, the rates of nondisjunction for these 3 genotypes have never been compared in isogenic strains, and the rates in the various combinations of double mutants have not been examined. In this study, we measure the rates of loss of chromosome I associated with these genotypes in isogenic diploids. In addition, we examine the frequency of loss and gain of all 16 chromosomes using CGH.

### System for detecting loss of chromosome I

All strains are isogenic except for alterations engineered by transformation. The wild-type diploid was constructed by a cross of the haploids MD761-5-16A and FY406 (details in *Materials and Methods*); these haploids are isogenic (FY406) or closely related (MD761-5-16A) to the canonical yeast strain S288c (Mortimer and Johnston 1986). In *S. cerevisiae*, there are 2 very similar genes that encode H2A (*HTA1* and *HTA2*) and H2B (*HTB1* and *HTB2*) (Wallis *et al*. 1980; Choe *et al*. 1982). In the diploids used in these experiments, the 2 chromosomal copies of the genes encoding H2A and H2B were deleted, and plasmids containing the wild-type copy of H2B and either a wild-type or mutant copy of H2A were used to maintain viability (Hirschhorn *et al*. 1995; Downs *et al*. 2000). The genotypes used in our analysis are shown in Supplementary File 1, and their construction is described in Supplementary Materials and Methods. In addition to mutations that affect chromosome nondisjunction, strains that contained the *mec1* mutation also were homozygous for the *sml1* mutation. This mutation suppresses the lethality associated with deletions of *mec1* (Zhao *et al*. 1998).

All of the diploids used to monitor chromosome loss were heterozygous for an insertion of *URA3* near the centromere of chromosome I. Loss or inactivation of the wild-type *URA3* gene results in a strain that is resistant to 5-FOA (Boeke *et al*. 1987). Thus, by measuring the frequency of 5-FOA^R^ derivatives in >20 independent cultures and converting the frequencies into a rate estimate using the method of the median (Lea and Coulson 1949), we could estimate the rate of chromosome I loss (discussion below).

**Fig. 1.**
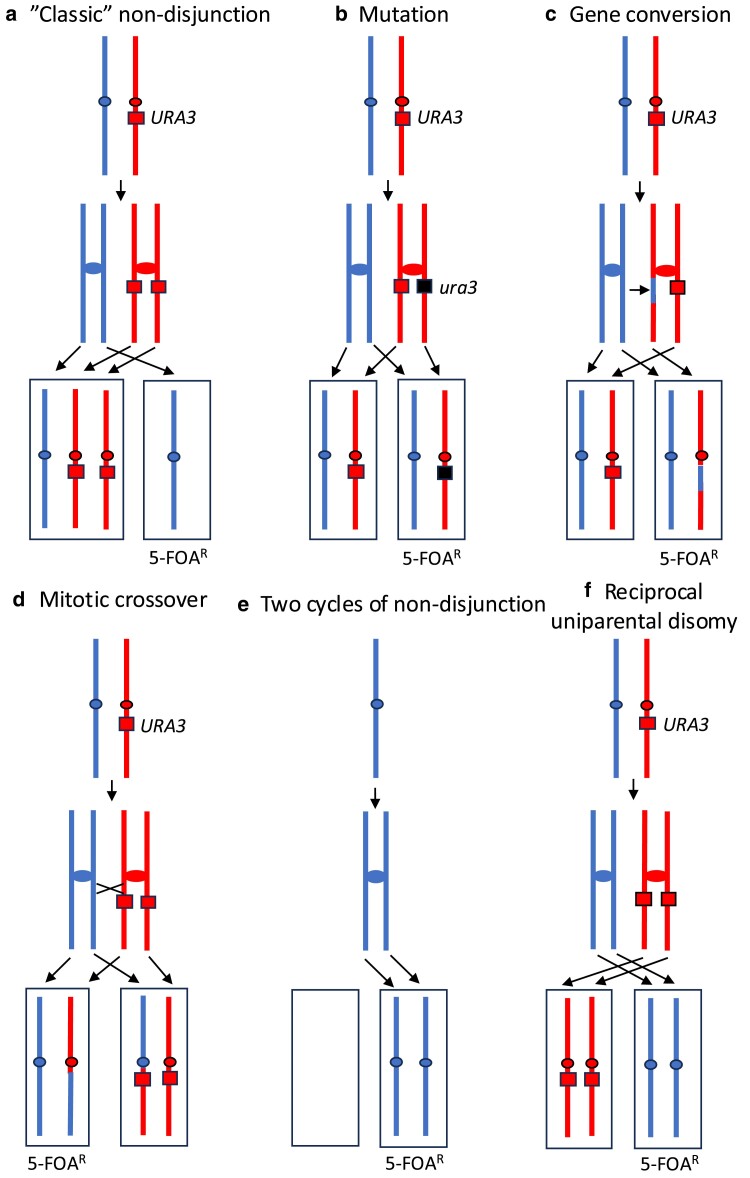
Mechanisms that result in 5-FOA resistance in a diploid heterozygous for a centromere-associated *URA3* marker. In this figure, blue and red colors represent the chromosome I homologs derived from the haploids MD761-5-16A and FY406 (Supplementary File 1), respectively. The *URA3* insertion is close to the centromere on the FY406-derived homolog. 5-FOA-resistant derivatives of the heterozygous parental diploid can arise by a number of different mechanisms. a) Classic nondisjunction. As a result of a nondisjunction event, 1 daughter cell is trisomic and the other is monosomic (5-FOA-resistant). b) Mutation. A mutation in the *URA3* gene (shown as a black rectangle) can produce a euploid 5-FOA-resistant derivative. c) Gene conversion. A double-strand break in the red homolog near the *URA3* insertion can result in replacement of the *URA3* gene with a segment of the blue homolog. d) Mitotic crossover. A mitotic crossover between the *URA3* gene and the centromere can result in 1 daughter that lacks the *URA3* gene and a second daughter that has 2 copies. A nonreciprocal recombination event (break-induced replication; Liu and Malkova 2022) can also result in a daughter cell lacking the *URA3* insertion. e) Two cycles of nondisjunction. One pathway that generates a 5-FOA^R^ isolate with 2 copies of the homolog is 2 consecutive nondisjunction events, the first producing a monosome and the second resulting in restoration of 2 copies of the homolog. f) RUPD. In this pathway, the chromosomes undergo a meiosis I-like division, resulting in 2 daughter cells with RUPD (Andersen and Petes 2012).

Although we expected that the 5-FOA^R^ isolates were a consequence of “classical” nondisjunction for chromosome I (Fig. 1a), other mechanisms could also produce a 5-FOA^R^ isolate (Fig. 1b–f). A mutation in the wild-type copy of *URA3* is one alternative (Fig. 1b). A mitotic gene conversion (Fig. 1c) or a mitotic crossover (Fig. 1d) could also produce a 5-FOA^R^ isolate. Finally, 5-FOA^R^ derivatives could be produced by double nondisjunction events (Fig. 1e) or a nondisjunction event that results in reciprocal uniparental disomy (RUPD; Fig. 1f).

We did several subsequent experiments that supported the conclusion that most of the 5-FOA^R^ isolates were a consequence of chromosome nondisjunction rather than the other mechanisms shown in Fig. 1. First, we determined the copy number of each homolog in each isolate by CGH microarrays (details in *Materials and Methods*). In 107 of the 5-FOA^R^ derivatives examined in Table 1, 80 (75%) contained only 1 copy of chromosome I. The data supporting this conclusion are in Supplementary Table 1.

**Table 1.** Rates of chromosome I loss in strains of various genotypes.

Strain name	Relevant genotype	Rate of 5-FOA^R^/cell division (95% CL)	Rate relative to wild type
MD842-1 MD842-2	Wild-type H2A (*HTA1*)*^a^*	3.5 × 10^−6^ (2.8-4.4 × 10^−6^) 2.3 × 10^−6^ (2.0-5.0 × 10^−6^) Average: 2.9 × 10^−6^	1
MD850-1 MD852-1	*HTA1 sml1*	3.5 × 10^−6^ (2.4–6.0 × 10^−6^) 2.8 × 10^−6^ (1.9–4.2 × 10^−6^) Average: 3.2 × 10^−6^	1.1
MD844-2 MD844-3 MD845-1	*hta1-S122A^b^*	1.1 × 10^−3^ (0.7–1.4 × 10^−3^) 0.9 × 10^−3^ (0.7–1.4 × 10^−3^) 1.5 × 10^−3^ (0.8–1.6 × 10^−3^) Average: 1.2 × 10^−3^	413
MD851-1 MD853-2 MD853-2	*hta1-S122A sml1*	1.1 × 10^−3^ (0.2–1.7 × 10^−3^) 1.2 × 10^−3^ (0.7–2.2 × 10^−3^) 0.9 × 10^−3^ (0.7–1.0 × 10^−3^) Average: 1.1 × 10^−3^	379
MD913-1 MD913-2	*hta1-S129A*	5.2 × 10^−6^ (3.9–7.2 × 10^−6^) 2.6 × 10^−6^ (2.3–3,1 × 10^−6^) Average: 3.9 × 10^−6^	1.3
MD865-1 MD868-2	*HTA1 bub1*	0.6 × 10^−3^ (0.5–0.75 × 10^−3^) 0.5 × 10^−3^ (0.41–1.0 × 10^−3^) Average: 0.55 × 10^−3^	190
MD915-1 MD915-1 MD915-2 MD915-2	*HTA1 tel1 mec1 sml1*	0.3 × 10^−3^ (0.17–0.5 × 10^−3^) 0.29 × 10^−3^ (0.09–0.5 × 10^−3^) 0.25 × 10^−3^ (0.14–0.42 × 10^−3^) 0.15 × 10^−3^ (0.12–0.3 × 10^−3^) Average: 0.25 × 10^−3^	86
MD914-1 MD914-2	*hta1-S122A hta1-S129A*	0.6 × 10^−3^ (0.36–2.1 × 10^−3^) 1.4 × 10^−3^ (0.39–1.9 × 10^−3^) Average: 1.0 × 10^−3^	344
SGK663 SGK659 SGK659	*hta1-S122A bub1*	0.81 × 10^−3^ (0.68–1.11 × 10^−3^) 0.86 × 10^−3^ (0.34–2.33 × 10^−3^) 0.90 × 10^−3^ (0.6–1.26 × 10^−3^) Average: 0.86 × 10^−3^	295
MD917-1 MD917-2 MD917-3 MD918-1 MD918-2 MD918-2	*hta1-S122A tel1 mec1 sml1*	1.1 × 10^−3^ (0.55–1.6 × 10^−3^) 1.7 × 10^−3^ (0.7-.2.0 × 10^−3^) 0.8 × 10^−3^ (0.5–1.0 × 10^−3^) 0.6 × 10^−3^ (0.2–1.4 × 10^−3^) 1.1 × 10^−3^ (1.0–1.7 × 10^−3^) 1.6 × 10^−3^ (1.2–2.7 × 10^−3^) Average: 1.15 × 10^−3^	397
MD882-2 MD883-1	*bub1 tel1 mec1 sml1*	2.4 × 10^−3^ (1.7–02.9 × 10^−3^) 1.5 × 10^−3^ (1.3–2.1 × 10^−3^) Average: 1.95 × 10^−3^	672
MD884-1 MD885-1	*hta1-S122A bub1 tel1 mec1 sml1*	2.2 × 10^−3^ (1.7->2 × 10^−3^) 2.2 × 10^−3^ (1.2–2.3 × 10^−3^) Average: 2.2 × 10^−3^	758

These strains were heterozygous for a *URA3* insertion tightly linked to *CEN1*. Except as noted, the *URA3* gene was inserted on the chromosome I derived from the isogenic S288c strain FY406. 5-FOA-resistant derivatives were primarily a consequence of loss of the *URA3*-containing homolog (details in the text). Rates were compared by the nonparametric Wilcoxon–Mann–Whitney test and the resulting *P*-values are in Supplementary Table 2. A summary of our conclusions for single-mutant strains (for our purposes, th*e tel1 mec1 sml1* genotype is counted as a single mutant): (1) Rates of chromosome loss for wild-type, *sml1*, and *hta1-S129A* strains are not significantly different from each other (*P*-values of >0.01). In addition, the rates for the wild-type, *sml1*, and *hta1-S129A* strains are significantly lower (*P* < 0.01) than any of the strains with the *hta1-S122A*, *bub1*, or *tel1 mec1 sml1* mutations.(2) Strains of the *tel1 mec1 sml1* genotype have significantly less chromosome loss than the *hta1-S122A* or *bub1* genotypes (*P* < 0.0001). (3) The *bub1 tel1 mec1 sml1* and *hta1-S122A bub1 tel1 mec1 sml1* strains have rates of chromosome loss that are higher (*P* < 0.02) than any other examined genotype but are not significantly different from each other (*P* = 0.28).

^*a*^Two other wild-type strains were examined, SGK178 and SGK179. In these strains (constructed described in Supplementary Materials and Methods), we did not determine which chromosome I homolog contained the *URA3* insertion. The rates of 5-FOA resistance were as follows: 5.5 × 10^−6^/division (SGK178) and 4.5 × 10^−6^/division (SGK179).

^*b*^Two other *hta1-S122A* strains were examined, MD821 and SGK177. In these strains (constructed described in Supplementary Materials and Methods), we did not determine which chromosome I homolog contained the *URA3* insertion. The rates of 5-FOA resistance were as follows: 1.3 × 10^−3^/division (MD821) and 1.2 × 10^−3^/division (SGK177).

In 23 of 107 5-FOA^R^ derivatives (21%), there were 2 copies of chromosome I. To exclude the possibility that isolates with 2 copies of chromosome I were a consequence of a mutation in *URA3* (Fig. 1b), we performed PCR using primers that could determine whether isolates were heterozygous for the *URA3* insertion on I (indicating the presence of a mutation within the insertion) or homozygous for loss of the *URA3* insertion (details in *Materials and Methods*). In all 5-FOA^R^ isolates with 2 copies of chromosome I examined (23 of 23), the *URA3* insertion was missing from both copies.

To determine whether the 5-FOA^R^ isolates with 2 copies of chromosome I lacking insertions of *URA3* were a consequence of mitotic recombination (Fig. 1c and d) or the mechanisms shown in Fig. 1e and f, we constructed isogenic derivatives of several of the strains shown in Table 1 that had an additional marker (*hyg*, a gene resulting in resistant to hygromycin) on the opposite arm of chromosome I from the *URA3* insertion. These derivatives were MD1004 (*hta1-S122A*; isogenic with MD844), MD1010 (*tel1mec1sml1*; isogenic with MD915), and MD1005 (*hta1-S122A tel1mec1sml1*; isogenic with MD917/MD918) (Table 1 and Supplementary Table 1). These strains were chosen because the progenitor strains had multiple 5-FOA^R^ isolates with 2 copies of chromosome I (Supplementary Table 1).

**Fig. 2.**
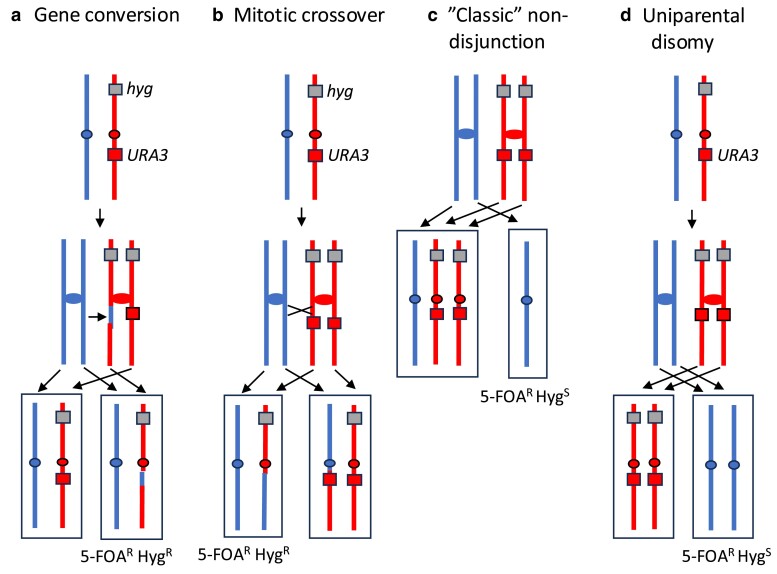
Analysis of genetic events producing 5-FOA^R^ derivatives utilizing diploids that contain a heterozygous *hyg* marker on chromosome I. In these diploids, the marker associated with resistance to hygromycin was placed on the opposite chromosome arm to the *URA3* gene. a) Gene conversion. A conversion event that deletes the URA3 gene can generate a 5-FOA^R^ isolate that is resistant to hygromycin. b) Mitotic crossover. Loss of the *URA3* marker as the result of a crossover between this marker and the centromere will result in a 5-FOA^R^ Hyg^R^ strain. c, d) These figures show 2 types of nondisjunction events that produce 5-FOA^R^ Hyg^S^ isolates, “classic” nondisjunction and RUPD. 5-FOA^R^ Hyg^S^ isolates can also result from 2 consecutive nondisjunction events (Fig. 1e).

As shown in Fig. 2a and b, if the loss of the *URA3* gene was a consequence of gene conversion or mitotic crossing over, then the 5-FOA^R^ diploid would be hygromycin-resistant (Hyg^R^) derivatives. In contrast, either of the nondisjunction pathways shown in Fig. 2c and d would produce hygromycin-sensitive (Hyg^S^) derivatives. Sixteen 5-FOA^R^ derivatives of MD1004 (*hta1-S122A*) were examined by microarrays, and 15 had 1 copy of chromosome I and one had 2 copies. All 16 strains were Hyg^S^. Thus, all 5-FOA ^R^ isolates were the result of nondisjunction by the pathways shown in Fig. 1a or 2c and d.

Of the 16 5-FOA^R^ derivatives of the *tel1mec1sml1* strain MD1010 examined by microarrays, 9 had 1 copy of chromosome I, 6 had 2 copies, and 1 had 3 copies. All derivatives with 1 copy of chromosome I were Hyg^S^ and, of the 7 strains with 2 or 3 copies of chromosome I, 4 were Hyg^S^ and 3 were Hyg^R^. In summary, most (13 of 16) of the 5-FOA^R^ isolates were the result of nondisjunction, although 3 were a consequence of mitotic recombination (Fig. 2a and b). Craven *et al*. (2002) previously showed that the *tel1mec1sml1* genotype elevated both mitotic recombination and chromosome loss by about 90-fold.

Of the 15 5-FOA^R^ derivatives of the *hta1-S122A tel1mec1sml1* strain MD1005, 12 had 1 copy of chromosome I and 3 had 2 copies. All 3 of the strains with 2 copies of chromosome I were Hyg^S^, indicating that these strains reflect one of the nondisjunction pathways shown in Figs. 1e and f and 2d. In summary, these results argue that the rates of 5-FOA resistance calculated in Table 1 are a good approximation of the rates of loss of chromosome I by nondisjunction. In summary, of 47 5-FOA^R^ strains derived from the *hta1-S122A*, *tel1mec1sml1*, and *hta1-S122A tel1mec1sml1* genotypes, 44 reflect chromosome loss.

### Rate of chromosome I loss in strains with histone mutations or other types of mutations that elevate the rate of aneuploidy (*bub1*, *tel1 mec1*)

Rates of chromosome I loss, as measured by the rates of 5-FOA^R^, were compared in mutant diploid strains to wild-type diploids (Table 1). The rates are determined for each strain by determining the frequency of 5-FOA^R^ in about 20 independent cultures per experiment and 2 independent experiments for each genotype. Frequencies were converted to rates using the method of Lea and Coulson (1949). The rates for the individual experiments are then averaged; 95% confidence intervals were calculated using Table B11 of Altman (1990). Significant differences between the rates observed in different strains were calculated by nonparametric Mann–Whitney tests (*Materials and Methods*), and the *P*-values associated these comparisons are in Supplementary Table 2; the footnote to Table 1 summarizes some of the most relevant comparisons.

The wild-type strain (MD842) had a rate of chromosome loss of 2.9 × 10^−6^/cell division. This rate is similar to that observed for loss of chromosome V (2 × 10^−6^/division) in a wild-type diploid of a different genetic background (Klein 2001). The diploids MD850/MD852 are genotypically identical to MD842 except these strains are homozygous for the *sml1* mutation. The Sml1 protein suppresses dNTP pools and mutations of *SML1* rescue the lethality associated with deletions of *MEC1* (Zhao *et al*. 1998). The rate of loss of chromosome I (3.2 × 10^−6^) was unaffected by the *sml1* deletion (Table 1).

As discussed in the Introduction, mutations of serine to alanine at either position 122 or 129 in *HTA1* result in sensitivity to methyl methanesulfonate. The phosphorylation of S129 is substantially reduced in *mec1* strains and is eliminated in strains of the *tel1mec1* genotype (Downs *et al*. 2000). S129, but not S122, is associated with the SQ motif characteristic of the targets of the Tel1 and Mec1 kinases. To investigate whether there was a connection between the phenotypes associated with S to A mutations of S122 and S129 and those associated with *tel1mec1* mutant strains, we examined the effects of various mutant combinations on chromosome loss. As shown in Table 1, an alanine substitution of S129 (*hta1-S129A*) had no significant effect on the rate of chromosome loss relative to the wild-type strain. In contrast, the rates of chromosome loss in the strains with the *hta1-S122A* substitution (MD844 and MD845) were elevated about 400-fold compared to the wild-type strain. A similar elevation was observed in strains with both the *hta1-S122A* and *sml1* mutations (MD851 and MD853).

Strains with a *bub1* mutation have substantially elevated rates of chromosome nondisjunction. The rate of loss of a small nonessential “test chromosome” in a haploid was elevated about 50-fold in a *bub1* deletion strain (Warren *et al*. 2002). The frequency of aneuploidy (production of both monosomes and trisomes) in a *bub1* diploid strain was at least 80-fold elevated compared to wild type (McCulley and Petes 2010). In our current analysis, the rates of chromosome I loss in *bub1* diploids (MD865 and MD868) were about 200-fold elevated compared to wild type (Table 1). This increase is similar, although smaller, to that observed in the strains with the *hta1-S122A* substitution, consistent with the hypothesis that phosphorylation of S122 by Bub1 is important to ensure accurate chromosome disjunction (Kawashima *et al*. 2010; Brimacombe *et al*. 2019). As discussed further below, it is possible that Bub1 is the main, but not the only kinase, capable of phosphorylating S122. It should also be pointed out that Bub1 has many potential targets for phosphorylation and *bub1* mutations affect several different cellular functions (for example, nonhomologous end-joining) that are not directly related to chromosome disjunction (Jessulat *et al*. 2015).

Strains of the *tel1mec1sml1* genotype also have high rates of chromosome loss. A *tel1mec1sml1* diploid had a chromosome V loss rate 89-fold above wild type (Craven *et al*. 2002). The frequencies of both monosomy and trisomy are elevated (McCulley and Petes 2010). In our current experiments, the rate of chromosome I loss was about 90-fold elevated (MD915; Table 1). The rate of chromosome loss in the *tel1mec1sml1* strain was about 4-fold less than observed in the *hta1-S122A* and *hta1-S122A sml1* strains. Although the rate estimates for the *bub1* strain and the *tel1mec1sml1* strain had overlapping 95% confidence intervals (Table 1), by a nonparametric test (described in Supplementary Materials and Methods), the *bub1* strain had a significantly higher rate of chromosome loss (about 2-fold) than the *tel1mec1sml1* strain (*P* < 0.001).

### Epistasis analysis of strains with high rates of chromosome loss

One method of determining whether gene products function in the same genetic pathway is epistasis analysis, determining the phenotype of double mutants compared to the phenotypes of the single mutants. One example of this type of analysis is the epistasis studies performed with yeast mutants sensitive to radiation (reviewed by Haynes and Kunz 1981). If the double mutant is no more sensitive to radiation than the single mutant with the strongest phenotype, the gene products are predicted to act in the same pathway. If the double mutant is much more sensitive to radiation than either single mutant (synergistic interaction), the 2 gene products are predicted to act on the same radiation-induced lesion in independent pathways. Lastly, if the double mutant has the sensitivity that is the sum of the sensitivities of the single mutants (additive interaction), one interpretation is that the gene products act independently on different radiation-induced lesions. Possible complications of these interpretations will be outlined in the *Discussion*.

We first examined the strain with the *hta1-S122A hta1-S129A* genotype. As expected, the rate of chromosome loss was approximately the same as those observed in the *hta1-S122A* and *hta1-S122A sml1* strains (Table 1). The rate of chromosome loss in the *hta1-S122A bub1* mutant was not significantly elevated compared to the single *hta1-S122A* and *bub1* mutants (Table 1). These results argue that both Bub1 and the serine 122-phosphorylated form of H2A function in the same pathway regulating chromosome segregation as suggested by Kawashima *et al*. (2010).

The rate in the strain with the genotype *hta1-S122A tel1mec1sml1* (1.2 × 10^−3^/cell division) was not significantly higher that the rates of chromosome loss in the *hta1-S122A* (1.2 × 10^−3^) or the *hta1-S122A sml1* strains. This result also implied that the effects of *tel1mec1* are exerted through phosphorylation of S122 on H2A. This interpretation, however, has 2 caveats. First, since S122 is not followed by a glutamine (the preferred target of Tel1 and Mec1), it is unlikely that S122 is a direct target of these kinases; in the *Discussion* section below and in Fig. 4, we suggest that Tel1 and Mec1 activate a different cellular kinase that is responsible for S122 phosphorylation. The second caveat is that, since the effect of the *tel1mec1sml1* mutation on chromosome loss is considerably less than the effect of *hta1-S122A*, we cannot exclude the possibility that the interaction of these mutations is additive. Additional details concerning the interactions of the *bub1*, *tel1/mec1*, and *hta1-S122A* mutations will be considered in the *Discussion*.

### Analysis of nondisjunction in unselected diploids using DNA microarrays

In our analysis thus far, we have emphasized the loss of chromosome I. This approach has 2 limitations. First, we examined only chromosome loss rates rather than chromosome gain events. There are mechanisms that result in chromosome loss other than chromosome nondisjunction. For example, diploid strains with a mutation in recombination protein *rad52* have very elevated rates of chromosome loss without chromosome gains (Mortimer *et al*. 1981; Song and Petes 2012), presumably because spontaneous double-strand DNA breaks that cannot be repaired efficiently result in chromosome loss. Second, our analysis was limited to one of the 16 yeast chromosomes.

To address these issues, we did 2 types of experiments. First, we examined a subset of strains with high rates of chromosome loss using CGH following multiple rounds of subculturing, We first analyzed strains of 5 genotypes (wild-type, *hta1-S129A*, *hta1-S122A*, *hta1-S122A hta1-S129A*, and *bub1*) that were subcultured from a single cell to a colony 5 times (total of 125 cell divisions); these strains did not have the heterozygous *URA3* insertion on chromosome I. We examined about 10 isolates per genotype.

**Fig. 3.**
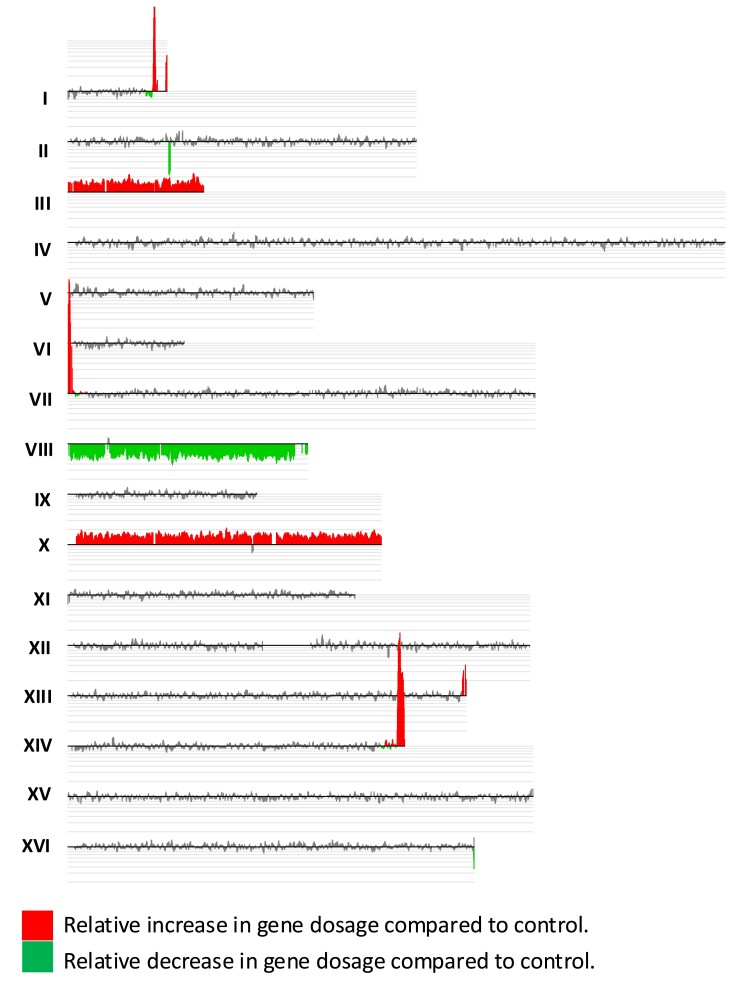
Example of CGH microarray from *hta1-S122A* strain (SGK177-9). The strain was a diploid formed by a cross of haploids isogenic with W303 and S288c. DNA isolated from the euploid control haploid W303 was labeled with Cy3 dUTP and the DNA from the experimental sample was labeled with Cy5 dUTP, and these samples were hybridized in competition to microarrays containing oligonucleotides that cover the entire yeast genome (details in *Materials and Methods*). The scanned microarray shows the ratio of hybridization for each chromosome. The red and green colors show significant relative increases and decreases of hybridization in the experimental sample vs the control sample. The light gray lines above each chromosome are log_2_ transforms of the ratio with the baseline having a value of 0, the first line above the baseline indicating a value of 1 and so on. Thus, euploid chromosomes have a log_2_ value of 0, trisomic chromosomes a log_2_ value of 0.6, and tetrasomic chromosomes a value of 1. The data of isolate SGK177-9 show that the experimental strain is monosomic for chromosome VIII and trisomic for chromosomes III and X. “Spikes” of the red signal are telomere-associated sequences present in the diploid starting strain that are absent in the control strain.

Figure 3 shows an example of the microarray analysis of an isolate from a strain of the *hta1-S122A* genotype after 5 cycles of subculturing with 1 subculturing defined as the growth of a single cell to a colony; we estimate about 25 cell divisions are required to form a colony (Sui *et al*. 2020). In Fig. 3, significant increases in hybridization (trisomy) relative to the parental diploid are shown in red, and significant decreases in hybridization (monosomy) are shown in green. Thus, the isolate in Fig. 3 is trisomic for chromosomes III and X and monosomic for chromosome VIII.

For each isolate, we determined the number of trisomic and monosomic strains that accumulated during the subculturing. We also examined the parental strain from which the subcultured isolates were derived. For strains with the *hta1-S122A*, *hta1-S122A*, *S129A*, and *bub1* genotypes, the parental strains before subculturing were each trisomic for 1 chromosome. Consequently, we did not include chromosomes that were aneuploid in the starting strain in our analysis. A summary of the aneuploidy in the isolates of each genotype is in Table 2.

**Table 2.** Aneuploidy in diploid strains of different genotypes subcultured 5 times as diagnosed by CGH.

Chromosome number	Chromosome changes in MD802 (wild-type)	Chromosome changes in MD806 (*hta1-S129A*)	Chromosome changes in MD808 (*hta1-S122A*)*^a^*	Chromosome changes in MD810 (*hta1-S122A, S129A*)*^b^*	Chromosome changes in MD816/MD817 (*bub1*)*^c^*
**I**	0	0	5 trisomes/2 monosomes	2 trisomes/3 monosomes	1 trisome/4 monosomes [1 trisome]
**II**	0	0	5 trisomes	3 trisomes	6 trisomes [1 trisome]
**III**	0	0	ND	3 trisomes	8 trisomes [ND]
**IV**	1 trisome	0	0	0	0 [0]
**V**	0	0	0	1 trisome/1 monosome	3 trisomes [0]
**VI**	0	0	0	0	0 [0]
**VII**	0	0	1 trisome	1 trisome	1 trisome [0]
**VIII**	0	0	4 trisomes	4 trisomes	5 trisomes [ND]
**IX**	0	1 trisome	1 monosome	5 monosomes	0 [0]
**X**	0	0	5 trisomes	ND	ND [1 trisome]
**XI**	0	0	1 trisome	4 monosomes	1 monosome [0]
**XII**	0	0	1 trisome	1 monosome	0 [0]
**XIII**	0	0	1 trisome	0	1 monosome [0]
**XIV**	0	0	1 trisome	1 monosome	1 monosome [1 trisome]
**XV**	0	0	1 trisome	1 trisome/2 monsomes	1 monosome [0]
**XVI**	0	0	8 trisomes	2 trisomes/1 monosome	4 trisomes [0]
**# of isolates**	11	10	11	11	10 MD416 [4 MD417]
**# of trisomes**	1	1	33	17	28 [4]
**# of monosomes**	0	0	3	18	8 [0]
**# of aneuploids**	1	1	36	35	36 [4]
**# of chromosomes analyzed*^d^***	352	320	330	330	300 [112]
**Frequency of aneuploids per** **chromosome*^e^***	2.8 × 10^−3^	3.1 × 10^−^	1.1 × 10^−1^	1.1 × 10^−1^	1.2 × 10^−1^ [3.6 × 10^−2^]
**Rate of aneuploids per cell division per chromosome*^f^***	2.3 × 10^−5^ (1)	2.5 × 10^−5^ (1.1)	8.7 × 10^−4^ (38)	8.7 × 10^−4^ (38)	9.6 × 10^−4^ [2.9 × 10^−4^] Average = 6.3 × 10^−4^ (27)

^*a*^MD808 before subcloning was trisomic for chromosome III, and, therefore, chromosome III was not included in the analysis (indicated by ND).

^*b*^MD808 before subcloning was trisomic for chromosome X, and, therefore, chromosome X was not included in the analysis.

^*c*^Two independently constructed isogenic strains were examined: MD816 (10 isolates) and MD817 (4 isolates). MD816 before subcloning was trisomic for chromosome X and MD817 was trisomic for chromosomes III and VIII. Data from MD817 are shown in brackets.

^*d*^The number of chromosomes analyzed is equal to the number of isolates times the number of chromosomes in the diploid isolate, excluding those chromosomes that were present as extra chromosomes in the strain before subcloning.

^*e*^The frequency of aneuploids per chromosome equals the number of aneuploid events (trisomy + monosomy) divided by the total number of chromosomes assayed.

^*f*^The rate of aneuploids per cell division per chromosome was calculated by dividing the frequency of aneuploids per chromosome by the number of cell divisions (estimated as 125). For the *bub1* strains MD816 and MD817, the rates were averaged. The rates relative to the wild-type rate are shown in parentheses.

To calculate the rate of aneuploidy per genome per cell division, we divided the sum of de novo chromosome loss and gain events by the total number of chromosomes examined (excluding those chromosomes that were aneuploid before subculturing); this number is the frequency of aneuploidy per chromosome. This number was divided by the number of cell divisions (125, 25 cell divisions/subculturing × 5 subculturings) to give the rate of aneuploidy per cell division per chromosome. For example, not counting alterations of chromosomes in the starting strain, we found 36 aneuploid chromosomes in a sample of 330 chromosomes (frequency of aneuploidy per chromosome of 0.11) in MD808 (*hta1-S122A* strain). Dividing this frequency by 125 (the number of cell divisions), we obtain a rate of aneuploidy per chromosome per cell division of 8.7 × 10^−4^; this calculation assumes that all chromosomes have an equal probability of nondisjunction. Similar calculations were done for the other 4 strains (Table 2).

There are several conclusions from this analysis. First, as expected from our previous analysis of loss of chromosome I, strains with the *hta1-S122A*, *hta1-S122A S129A*, and *bub1* genotypes have much higher (about 40-fold) rates of aneuploidy than the wild-type and *hta1-S129A* strains. Second, in addition to chromosome losses, there are frequent chromosome gains. Thus, the high rate of chromosome I loss detected in our studies likely involves chromosome nondisjunction rather than loss based on the other mechanisms described above. Although we observed both duplications (leading to trisomy) and deletions (leading to monosomy), trisomes outnumbered monosomes by a factor of 3. One likely explanation of this observation is that trisomic strains have less of a growth defect than monosomic strains, since about 200 yeast genes are haplo-insufficient (Deutschbauer *et al*. 2005). Third, the microarray studies confirm the importance of phosphorylation of S122 of histone H2A and the contrasting lack of importance of S129 phosphorylation in regulating chromosome disjunction.

### Microarray analysis of diploid strains selected to be monosomic for chromosome I

We also performed microarray analysis on a subset of the diploid strains selected to be 5-FOA resistant (Supplementary Table 1). Approximately 10 5-FOA-resistant independent isolates were examined for each genotype. We calculated the approximate rate of loss of unselected chromosomes in each strain by first determining the number of aneuploid chromosomes, excluding the selected chromosome (chromosome I) as well as chromosomes that were aneuploid in all isolates (which likely reflect chromosomes that were aneuploidy before selection on 5-FOA). This number was divided by the total number of chromosomes to determine the frequency of aneuploidy per chromosome. Since ∼25 cell divisions are required to form a colony from a single cell, we divided the frequency of aneuploidy/chromosome by 25 to get an approximate rate of aneuploidy per chromosome per cell division.

The rates of aneuploidy per chromosome per cell division were <3 × 10^−4^ or less (wild-type, *hta1-S129A*, and *sml1*) or >2 × 10^−3^ (*hta1-S122A*, *hta1-S122A hta1-S129A*, *bub1*, *mec1tel1sml1*, *mec1tel1sml1hta1-S122A*, *mec1tel1sml1bub1*, *mec1tel1sml1bub1hta1-S122A*). Although these rate measurements are in qualitative agreement with the rates estimated in other types of experiments (Tables 1 and 2), there are quantitative differences that will be discussed below.

### Comparisons of rate estimates in different assays for aneuploidy

We used 3 different approaches are measuring the rates of aneuploidy in strains of different genotypes: assay 1: Measurement of the rate of loss of chromosome I (rate of 5-FOA^R^ derivatives; Table 1) using many (>20) independent cultures of each strain; assay 2: measurement of the rate of aneuploidy (gain and loss) of yeast chromosomes in unselected yeast strains following 125 cell divisions using DNA microarrays (Table 2); and assay 3: measurement of the rate of aneuploidy of all yeast chromosomes in strains that have lost chromosome I. In all 3 assays, the wild-type strain and strain with the *hta1-S129A* genotype have very low rates of nondisjunction, whereas strains with the *hta1-S122A*, *bub1*, and *hta1-S122A hta1-S129A* genotypes have rates that are elevated 10- to several hundred-fold above the wild-type rate. In addition, in the analyses done with assay 1 and assay 3 (not performed with assay 2), the strains with the genotypes *tel1mec1sml1*, *hta1-S122A tel1mec1sml1*, *bub1tel1mec1sml1*, and *hta1-S122A bub1tel1mec1sml1* had very high rates of instability (Table 1 and Supplementary Table 2).

**Fig. 4.**
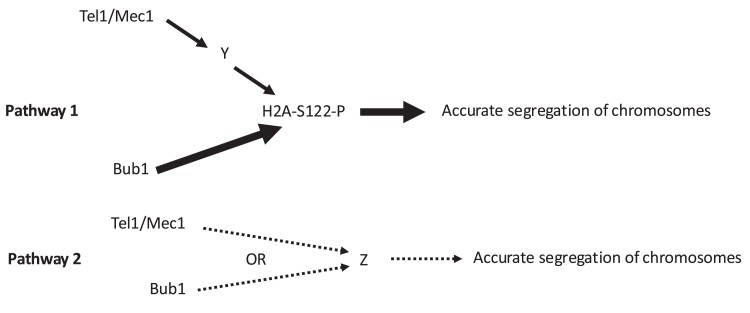
Model that summarizes the regulation of chromosome disjunction by the genes examined in our study. We suggest that 2 pathways are regulated by these genes. In pathway 1, the phosphorylation of H2A on serine 122 is important for accurate chromosome segregation with Bub1 having a direct role in this phosphorylation and Tel1 having an indirect minor role. In pathway 2, Tel1 and Mec1 are functionally redundant, phosphorylating an unknown target (Z) that regulates chromosome segregation. Thus, strains with blocks in both pathways (genotypes *tel1mec1sml1bub1* or *tel1mec1sml1hta1-S122A*) have the highest level of chromosome nondisjunction. The relative width of the arrows in pathway 1 indicates that Bub1 is the major kinase that phosphorylates S122 of H2A with a Tel1/Mec1-dependent pathway having a smaller role. The dotted lines in pathway 2 indicate that this pathway is based solely on the epistasis analysis and, in addition, is a back-up pathway.

In comparing the estimates of aneuploidy obtained with the 3 different approaches, a number of points should be stressed. First, the rate measurements obtained from assay 1 are much more accurate than the rates estimated by the other techniques, since these rate estimates are based on analyzing many independent colonies each of which has multiple 5-FOA^R^ isolates. In contrast, the rate estimates based on the use of microarrays often involve few aneuploid isolates. For example, the rate of aneuploidy calculated for the wild-type strain MD802 (assay 2) was based on a single aneuploid event (Table 2). Thus, our conclusions about the epistasis of various mutant combinations summarized in Fig. 4 are based entirely on the data shown in Table 1. Second, the rate estimates of assay 1 are based exclusively on loss of chromosome I whereas the other 2 assays examined both loss and gain of all of the chromosomes. Third, assay 2 involved 5 cycles of subcloning (∼125 cell divisions) whereas the other 2 assays did not involve subcloning. Since aneuploid strains grow more slowly than euploid strains (Torres *et al*. 2007) and this effect may be homolog specific, the subcloning may alter the observed patterns of aneuploidy. In assay 2, we found that 9 of the observed 29 monosomic strains in Table 2 were chromosome I monosomes, chromosome I being the smallest yeast chromosome. This proportion (0.31) is about 5-fold higher than the expected proportion if monosomic strains for all chromosomes were recovered with equal frequencies (0.06) (*P* < 0.001). Despite all of these caveats, the effects of the various mutants on chromosome disjunction are in good qualitative agreement for all 3 types of assays.

## Discussion

The process that ensures accurate mitotic chromosome segregation is complex, involving more than 100 proteins (McAinsh and Marston 2022). These proteins are required for multiple functions: complete replication of DNA, establishment of cohesion between the replicated sister chromatids, assembly of a large complex of kinetochore proteins on the centromeric DNA and bipolar attachment of microtubules onto the kinetochores. The correct attachment of microtubules to the kinetochore is detected by the spindle assembly checkpoint proteins. As expected from the large numbers of proteins involved in these functions, more than 500 genes when mutated elevate chromosome nondisjunction (Stirling *et al.* 2012). In this study, we examine the chromosome nondisjunction events that result from a mutation in histone H2A that prevents phosphorylation of a serine located at position 122. In isogenic genetic backgrounds, we compared chromosome loss events in other mutant strains (*bub1*, *tel1*, and *mec1*) with high levels of nondisjunction.

### Comparison with previous studies of the effects of mutations in histone H2A

Although not detected in large-scale screens, the *hta1-S122A* mutation was found to be responsible for high rates of chromosome nondisjunction in several other studies. In analysis of the comparable mutant (*h2a-SA*) in *S. pombe*, Kawashima *et al*. (2010) showed that this mutation elevated the rate of lagging chromosomes to a degree that was approximately the same as observed in *bub1* mutants. Since the same elevation was also observed in the *h2a-SA bub1* double mutant, Kawashima *et al.* argued that the 2 proteins acted in the same pathway. They also showed that Bub1 phosphorylated H2 serine on residue 121, equivalent to S122 in *S. cerevisiae*. Failure to phosphorylate H2A, either because of the *h2a-SA* mutation or a *bub1* mutation resulted in a defect in the recruitment of shugoshin to the kinetochore, causing a spindle assembly checkpoint defect. Similarly, strains of *C. albicans* that have an H2A histone that cannot be phosphorylated on S121 have a high rate of chromosome loss, roughly equivalent to loss rates in strains with *bub1* or *sgo1* mutations (Brimacombe *et al*. 2019). Lastly, the effects of mutating serine 122 of H2A in *S. cerevisiae* were examined using CGH microarrays (Uffenbeck 2013). In this study in haploid cells, the *hta1-S122A* mutation was associated with a higher rate (about 10-fold) of nondisjunction than observed in *bub1* strains.

In addition to the *hta1-S122A* mutation, 2 other mutations in H2A in *S. cerevisiae* are associated with high levels of nondisjunction: *hta1-200* (S20F substitution) and *hta1-300* (G30D substitution) (Pinto and Winston 2000). These mutations elevate chromosome gains in haploids about 100-fold and chromosome losses in diploids about 10- to 15-fold. Both mutations affect chromatin structure at the centromere. It will be of interest to determine whether the *hta1-S122A* mutation has similar effects on centromere chromatin. It is likely that there are other mutant forms of H2A that would have strong effects on chromosome disjunction. Because there are 2 genes encoding H2A (*HTA1* and *HTA2*), classical mutant hunts would likely fail to detect such mutations.

### 5-FOA-resistant derivatives of strains heterozygous for a centromere-linked *URA3* gene on chromosome I are usually monosomic for chromosome I

As described above, most of the 5-FOA^R^ derivatives of the strains with the centromere-linked heterozygous *URA3* marker were monosomic. There were 2 interesting exceptions. The *tel1mec1sml1* strain MD1010 had several isolates that were the result of mitotic recombination. This finding likely is a consequence of the 90-fold elevation in the rate of mitotic exchange in strains of this genotype (Craven *et al*. 2002).

The second surprising observation is that about 20% of the 5-FOA^R^ isolates of the strains examined retained 2 copies of chromosome I, both of which lacked the *URA3* insertion. These strains could be explained by multiple cycles of nondisjunction (Fig. 1e) or by reciprocal uniparental inheritance (RUPD; Fig. 1f). Since the frequency of these events is much higher than expected by 2 independent chromosome I nondisjunction events, we suggest that these isolates are probably the result of RUPD. We previously showed that RUPD, a meiosis I-like segregation in which 1 pair of the homolog is segregated into 1 daughter cell and the other pair is segregated into the second daughter, is often responsible for loss of heterozygous markers in yeast (Andersen and Petes 2012).

### Model of chromosome segregation based on the epistasis results

Our results and those of others (Kawashima *et al*. 2010; Uffenbeck 2013; Brimacombe *et al*. 2019) support the 2-pathway model shown in Fig. 4. We suggest that pathway 1 is the most important pathway with a relatively minor role for pathway 2. In pathway 1, we hypothesize that phosphorylation of serine 122 is critical for accurate chromosome segregation and the major kinase responsible for this phosphorylation is Bub1 as suggested by Kawashima *et al*. (2010). Since the rate of chromosome loss is greater in the *hta1-S122A* strain than in the *bub1* strain, the phosphorylation of S122 may also be partially dependent on the Tel1/Mec1 kinases. Since the S122 serine is not part of an SQ motif that is the preferred target for the Mec1 and Tel1 kinases (Lanz *et al*. 2019), it is likely that the postulated stimulatory effect of Tel1 and Mec1 on phosphorylation of S122 is indirect. The direct target of these kinases in H2A is S129. Failure to phosphorylate S129 results in sensitivity to the DNA-damaging agent methyl methanesulfonate (Downs *et al*. 2000) but has no effect on chromosome disjunction (present study).

Although the rate of chromosome loss was very high in strains with the *hta1-S122A* mutation, the highest rates of chromosome loss were observed in strains with the *bub1tel1mec1sml1* and *hta1-S122A bub1tel1mec1sml1* genotypes (Table 1). This observation indicates that the Bub1, Tel1, and Mec1 proteins may have effects on chromosome loss in addition to their effects on phosphorylation of S122A (pathway 2). We suggest that Bub1 and the Tel1/Mec1 kinases have an additional phosphorylation target in which they have redundant effects. It should be noted that Tel1 and Mec1 often phosphorylate the same substrate including, S129A of histone H2A (Downs *et al*. 2000), and other proteins (Mallory and Petes 2000). Given the large number of proteins required for centromere function and the spindle assembly checkpoint, the number of potential substrates of these kinases is very large.

### Caveats concerning the estimates of chromosome loss

Some of the strains with *bub1*, *hta1-S122A*, or *tel1mec1* mutations were aneuploid before isolating 5-FOA^R^ derivatives (Supplementary Table 1). It is unlikely, however, that this preexisting aneuploid substantially affected subsequent chromosome loss rates. We often analyzed patterns of aneuploidy in more than 1 derivative of a specific genotype (Supplementary Table 1). Although these derivatives sometimes had different patterns of aneuploidy (or no aneuploidy) in the starting strain, the levels of de novo aneuploidy were often similar. For example, we examined 2 isogenic strains with the *hta1-S122A* mutation, MD821 (no aneuploidy in the starting strain) and SGK177 (trisomy for chromosome X). The frequencies of aneuploidy in the 2 strains were 3/isolate (24 aneuploids/8 isolates in MD821) and 2/isolate (20 aneuploids/10 isolates in SGK177). In strains with th*e tel1mec1sml1* mutations (MD915-1 and MD915-2), MD915-1 had no aneuploid events in the starting strain whereas MD915-2 was trisomic for chromosomes XI and XII. The frequencies of aneuploidy between these 2 strains were similar, 2.25 (9 aneuploids/4 isolates in MD915-1) and 1.5 (6 aneuploids/4 isolates in MD915-2). Although these frequencies are based on a small number of events, the results in Supplementary Table 1 argue that the aneuploids existing in some of the starting strains do not have a large effect on the observed rates of aneuploidy.

Another potential caveat is that aneuploidy for specific chromosomes could compensate for slow growth of some of the mutant strains. This possibility cannot be excluded, although most of the mutants had no obvious growth defect except for strains with the *tel1mec1sml1* genotype. In a previous study (Vernon *et al.* 2008), we found that haploid *S. cerevisiae* strains with the hypomorphic mutation *mec1-21* often became disomic for chromosome VIII; this property was suppressed by transformation of a plasmid with the *DNA2* gene, a DNA helicase/endonuclease involved in DNA replication and telomere maintenance. In our study, strains of the *mec1tel1sml1* genotype did not frequently become trisomic for chromosome VIII although monosomy of this chromosome was common (Supplementary Table 1). The difference in our current observations and those of Vernon *et al.* may reflect the use of diploids in our current study vs haploids used in the previous study, the difference between the null *mec1* allele in the current study vs the hypomorphic allele used in the current study or other factors.

In part, because of the caveats discussed above, we stress that the model shown in Fig. 4, although consistent with our data, is highly speculative. Identification of the postulated kinase shown in pathway 2 would considerably strengthen our inferences.

## Conclusions

- Serine-to-alanine mutations at position 122 of histone H2A lead to very elevated rates of chromosome nondisjunction. Although many different homologs were observed as aneuploids (Supplementary Table 1), the distribution of aneuploidy was uneven. For example, in the 51 strains with the *hta1-S122A* mutation (by itself or in combination with other mutations), we observed 19 aneuploid events. For the same 51 strains, no such events were observed for chromosome IV (the largest chromosome) or VI. The lack of such events may be the result of poor growth in strains monosomic for chromosomes IV and VI due to genes on these chromosomes that affect growth when overexpressed or underexpressed
- Mutations of serine to alanine at position 129 of histone H2A have no effect on chromosome nondisjunction.
- Our observations of the rate of nondisjunction caused by the *bub1* mutation are consistent with previous observations that Bub1 phosphorylates H2A on serine 122, and this phosphorylation is important for accurate chromosome segregation.
- In addition to phosphorylating histone H2A, the epistasis analysis suggests that Bub1, Tel1, and Mec1 act in a second pathway required for accurate chromosome segregation that is independent of phosphorylation of H2A.
- Uniparental disomy is common in strains with the *hta1-S122A* mutation.

## Supplementary Material

iyae194_Supplementary_Data

## Data Availability

Plasmids and strains used in our study are available on request. The authors state that the data necessary to evaluate our conclusions are provided within the article or in the Supplementary material, which is available at Genetics online. Supplemental material available at GENETICS online.
