# Supplementary material for: A tale of two serines: the effects of histone H2A mutations S122A and S129A on chromosome nondisjunction in Saccharomyces cerevisiae
Source: Genetics. 2024 Nov 18;229(1):iyae194. doi: 10.1093/genetics/iyae194 (PMC11708911; doi:10.1093/genetics/iyae194)
Supplement: iyae194_Supplementary_Data [file iyae194_supplementary_data.zip › Supplemental_File_S1_GENETICS-2024-307465.docx]

**Supplemental File S1. Genotypes of yeast strains used in the study.**

**Haploid strains (*MAT*a).**

**S288c.** Wild-type *MAT***a** haploid (<https://www.yeastgenome.org/strain/s288c>),

**SGK42.** *MAT***a** *lys2*Δ::*Hyg.*

**SGK80.** *MAT***a** *lys2*Δ::*Hyg ura3*Δ::*Kan*

**FY602** (provided by Fred Winston, Harvard Medical School; https://winston-strain-finder.hms.harvard.edu/yeast/12493). *MAT***a** *ura3-52 leu2-∆1 lys2-128∆ his3-∆200 trp1-∆63*

**MD813.** *MAT***a** *ura3-52 leu2∆1 lys2-128∆ his3∆200 trp1∆63 bub1∆::Hyg.*

**FY406.** *MAT***a** *(hta1-htb1)∆::LEU2 (hta2-htb2)∆::TRP1 ura3-52 leu2∆1 lys2-128∆ his3∆200 trp1∆63* pAB6 *(HTA1-HTB1; URA3)*; pAB6 contains the wild-type *HTA1* and *HTB1* genes in the centromere-containing vector pRS316 (Hirschhorn *et al.*, 1995).

**FY406-pJD150-HIS3.** *MAT***a** *(hta1-htb1)∆::LEU2 (hta2-htb2)∆::TRP1 ura3-52 leu2∆1 lys2-128∆ his3∆200 trp1∆63* pAB6 *(HTA1-HTB1; URA3)* pJD150-HIS3 *(HTA1-HTB1; HIS3 )*.

**SGK330/SGK331.** *MAT***a** *(hta1-htb1)∆::LEU2 (hta2-htb2)∆::TRP1 ura3-52 leu2∆1 lys2-128∆ his3∆200 trp1∆63* pJD150-HIS3 *(HTA1-HTB1; HIS3 )*.

**SGK334/SGK336:** *MAT***a** *(hta1-htb1)∆::LEU2 (hta2-htb2)∆::TRP1 ura3-52 leu2∆1 lys2-128∆ his3∆200 trp1∆63* pJD150-HIS3 *(HTA1-HTB1; HIS3) CEN1::URA3.*

**MD825-1,2/MD826-1,2.** *MAT***a** *(hta1-htb1)∆::LEU2 (hta2-htb2)∆::TRP1 ura3-52 leu2∆1 lys2-128∆ his3∆200 trp1∆63* pJD150-HIS3 *(HTA1-HTB1; HIS3) CEN1::URA3 sml1∆::loxP-Hyg-loxP.*

**MD863.** *MAT***a** *(hta1-htb1)∆::LEU2 (hta2-htb2)∆::TRP1 ura3-52 leu2∆1 lys2-128∆ his3∆200 trp1∆63* pJD150-HIS3 *(HTA1-HTB1; HIS3) CEN1::URA3 sml1∆::loxP-Nat-loxP.*

**MD829-1/MD830-1:** *MAT***a** *(hta1-htb1)∆::LEU2 (hta2-htb2)∆::TRP1 ura3-52 leu2∆1 lys2-128∆ his3∆200 trp1∆63 CEN1::URA3 sml1Δ::loxP-Hyg-loxP)* pJD150-Kan *(HTA1-HTB1; KanMX).*

**TDP4:** *MAT***a** *(hta1-htb1)∆::LEU2 (hta2-htb2)∆::TRP1 ura3-52 leu2∆1 lys2-128∆ his3∆200 trp1∆63* pJD150-HIS3 *(HTA1-HTB1; HIS3) CEN1::URA3* pJD150-Kan *(HTA1-HTB1; KanMX)*.

**MD833-1,2/MD834-1,2:** *MAT***a** *(hta1-htb1)∆::LEU2 (hta2-htb2)∆::TRP1 ura3-52 leu2∆1 lys2-128∆ his3∆200 trp1∆63 CEN1::URA3* pJD150-Kan *(HTA1-HTB1; KanMX)*.

**MD1000-1.** *MAT***a** *(hta1-htb1)∆::LEU2 (hta2-htb2)∆::TRP1 ura3-52 leu2∆1 lys2-128∆ his3∆200 trp1∆63 CEN1::URA3 I-9500::Hyg* pJD150-Kan *(HTA1-HTB1; KanMX)*.

**MD1001-1.** *MAT***a** *(hta1-htb1)∆::LEU2 (hta2-htb2)∆::TRP1 ura3-52 leu2∆1 lys2-128∆ his3∆200 trp1∆63 CEN1::URA3 I-9500::Hyg sml1Δ::loxP* pJD150-Kan *[HTA1-HTB1; KanMX) tel1Δ::loxP* p*MEC1-BUB1-LYS2 mec1Δ::Nat.*

**MD858-1,2/MD859-1,2.** *MAT***a** *(hta1-htb1)∆::LEU2 (hta2-htb2)∆::TRP1 ura3-52 leu2∆1 lys2-128∆ his3∆200 trp1∆63 CEN1::URA3* pJD150-Kan *(HTA1-HTB1; KanMX)* pMEC1-BUB1-LYS2 *bub1Δ::Hyg.*

**FY406-pJD151.** *MAT***a** *(hta1-htb1)∆::LEU2 (hta2-htb2)∆::TRP1 ura3-52 leu2∆1 lys2-128∆ his3∆200 trp1∆63* pAB6 *(HTA1-HTB1; URA3)* pJD151 *(hta1-S129A-HTB1; HIS3)*.

**FY406-pJD190.** *MAT***a** *(hta1-htb1)∆::LEU2 (hta2-htb2)∆::TRP1 ura3-52 leu2∆1 lys2-128∆ his3∆200 trp1∆63* pAB6 *(HTA1-HTB1; URA3)* pJD190 *(hta1-S121A-HTB1; HIS3)*.

**FY406-pJD197.** *MAT***a** *(hta1-htb1)∆::LEU2 (hta2-htb2)∆::TRP1 ura3-52 leu2∆1 lys2-128∆ his3∆200 trp1∆63* pAB6 *(HTA1-HTB1; URA3)* pJD197 *(hta1-S121A,S128A-HTB1; HIS3)*.

**MD871-1,2.** *MAT***a** *(hta1-htb1)∆::LEU2 (hta2-htb2)∆::TRP1 ura3-52 leu2∆1 lys2-128∆ his3∆200 trp1∆63 CEN1::URA3 sml1Δ::loxP-Hyg-loxP* pJD150-Kan *[HTA1-HTB1; KanMX] ) tel1Δ::loxP-Nat-loxP*

**MD871-1,2 + pSH47-LYS2(Cre).** *MAT***a** *(hta1-htb1)∆::LEU2 (hta2-htb2)∆::TRP1 ura3-52 leu2∆1 lys2-128∆ his3∆200 trp1∆63 CEN1::URA3 sml1Δ::loxP* pJD150-Kan *[HTA1-HTB1; KanMX) tel1Δ::loxP* pSH47-LYS2(Cre).

**MD873-1,2.** *MAT***a** *(hta1-htb1)∆::LEU2 (hta2-htb2)∆::TRP1 ura3-52 leu2∆1 lys2-128∆ his3∆200 trp1∆63 CEN1::URA3 sml1Δ::loxP* pJD150-Kan *[HTA1-HTB1; KanMX) tel1Δ::loxP*

**MD875-1,2.** *MAT***a** *(hta1-htb1)∆::LEU2 (hta2-htb2)∆::TRP1 ura3-52 leu2∆1 lys2-128∆ his3∆200 trp1∆63 CEN1::URA3 sml1Δ::loxP* pJD150-Kan *[HTA1-HTB1; KanMX) tel1Δ::loxP* p*MEC1-BUB1-LYS2 mec1Δ::Nat*

**MD879-1,2,3,4:** *MAT***a** *(hta1-htb1)∆::LEU2 (hta2-htb2)∆::TRP1 ura3-52 leu2∆1 lys2-128∆ his3∆200 trp1∆63 CEN1::URA3 sml1Δ::loxP* pJD150-Kan *[HTA1-HTB1; KanMX) tel1Δ::loxP* p*MEC1-BUB1-LYS2 mec1Δ::Nat bub1Δ::Hyg*

**Haploid strains (*MATα)***

**MD761-5-16A.** *MAT*α *(hta1-htb1)∆::LEU2 (hta2-htb2)∆::TRP1 leu2 his3 ura3 lys2-128Δ trp1 can1-100 pAB6 (HTA1-HTB1; URA3).*

**MD812-1,2.** *MAT*α *(hta1-htb1)∆::LEU2 (hta2-htb2)∆::TRP1 leu2 his3 ura3 lys2-128Δ trp1 can1-100* pAB6 *(HTA1-HTB1; URA3) bub1Δ::Hyg.*

**MD835-1,2.** *MAT*α *(hta1-htb1)∆::LEU2 (hta2-htb2)∆::TRP1 leu2 his3 ura3 lys2-128Δ trp1 can1-100* pJD150-Kan *(HTA1-HTB1; KanMX).*

**MD860-1,2,3.** *MAT*α *(hta1-htb1)∆::LEU2 (hta2-htb2)∆::TRP1 leu2 his3 ura3 lys2-128Δ trp1 can1-100* pJD150-Kan *(HTA1-HTB1; KanMX)* p*MEC1-BUB1-LYS2 bub1Δ::Hyg).*

**MD827-1,2:** *MAT*α *(hta1-htb1)∆::LEU2 (hta2-htb2)∆::TRP1 leu2 his3 ura3 lys2-128Δ trp1 can1-100 pAB6 (HTA1-HTB1; URA3) sml1∆::loxP-Hyg-loxP.*

**MD828-1,2.** *MAT*α *(hta1-htb1)∆::LEU2 (hta2-htb2)∆::TRP1 leu2 his3 ura3 lys2-128Δ trp1 can1-100 sml1∆::loxP-Hyg-loxP* pJD150-Kan *(HTA1-HTB1; KanMX).*

**MD870-1,2.** *MAT*α *(hta1-htb1)∆::LEU2 (hta2-htb2)∆::TRP1 leu2 his3 ura3 lys2-128Δ trp1 can1-100 sml1∆::loxP-Hyg-loxP* pJD150-Kan *(HTA1-HTB1; KanMX) tel1Δ::loxP-Nat-loxP.*

**MD870-1,2 + pSH47-LYS2(Cre).** *MAT*α *(hta1-htb1)∆::LEU2 (hta2-htb2)∆::TRP1 leu2 his3 ura3 lys2-128Δ trp1 can1-100 sml1∆::loxP* pJD150-Kan *(HTA1-HTB1; KanMX) tel1Δ::loxP* pSH47-LYS2(Cre)*.*

**MD872-1,2.** *MAT*α *(hta1-htb1)∆::LEU2 (hta2-htb2)∆::TRP1 leu2 his3 ura3 lys2-128Δ trp1 can1-100 sml1∆:: loxP* pJD150-Kan *(HTA1-HTB1; KanMX) tel1Δ::loxP.*

**MD874-1,2.** *MAT*α *(hta1-htb1)∆::LEU2 (hta2-htb2)∆::TRP1 leu2 his3 ura3 lys2-128Δ trp1 can1-100 sml1∆::loxP* pJD150-Kan *(HTA1-HTB1; KanMX) tel1Δ::loxP mec1Δ::Nat* pMEC1-BUB1-LYS2*.*

**MD878-1,2,3.** *MAT*α *(hta1-htb1)∆::LEU2 (hta2-htb2)∆::TRP1 leu2 his3 ura3 lys2-128Δ trp1 can1-100 sml1∆::loxP* pJD150-Kan *(HTA1-HTB1; KanMX) tel1Δ::loxP mec1Δ::Nat* pMEC1-BUB1-LYS2 *bub1Δ::Hyg.*

**RCY278-5A.** *MATα leu2-3,112 his3-11,15 ura3-1 ade2-1 trp1-1 can1-100 RAD5.*

**Diploid strains.**

**MD761-5.** *MAT***a***/MAT*α *(hta1-htb1)∆::LEU2/(hta1-htb1)∆::LEU2 (hta2-htb2)∆::TRP1/(hta2-htb2)∆::TRP1 ura3-52/ura3 leu2∆1/leu2 lys2-128∆/ lys2-128∆ his3∆200/his3 trp1∆63/trp1 CAN1/can1-100 pAB6 (HTA1-HTB1; URA3).*

**MD801.** *MAT***a/***MAT*α *(hta1-htb1)∆::LEU2/hta1-htb1)∆::LEU2 (hta2-htb2)∆::TRP1/ (hta2-htb2)∆::TRP1 ura3-52/ura3 leu2∆/leu2 lys2-128∆/ lys2-128Δ his3∆200/his3 trp1∆63/trp1 CAN1/can1-100 pAB6 (HTA1-HTB1; URA3)* pJD150-HIS3 (*HIS3*-containing centromeric plasmid with *HTA1-HTB1)*.

**MD802.** *MAT***a/***MAT*α *(hta1-htb1)∆::LEU2/hta1-htb1)∆::LEU2 (hta2-htb2)∆::TRP1/ (hta2-htb2)∆::TRP1 ura3-52/ura3 leu2∆/leu2 lys2-128∆/ lys2-128Δ his3∆200/his3 trp1∆63/trp1 CAN1/can1-100* pJD150-HIS3 (*HIS3*-containing centromeric plasmid with *HTA1-HTB1)*.

**SGK178/SGK179.** *MAT***a/***MAT*α *(hta1-htb1)∆::LEU2/hta1-htb1)∆::LEU2 (hta2-htb2)∆::TRP1/ (hta2-htb2)∆::TRP1 ura3-52/ura3 leu2∆/leu2 lys2-128∆/ lys2-128Δ his3∆200/his3 trp1∆63/trp1 CEN1::URA3/CEN1 CAN1/can1-100* pJD150-HIS3 (*HIS3*-containing centromeric plasmid with *HTA1-HTB1)*.

**MD821.** *MAT***a/***MAT*α *(hta1-htb1)∆::LEU2/hta1-htb1)∆::LEU2 (hta2-htb2)∆::TRP1/ (hta2-htb2)∆::TRP1 ura3-52/ura3 leu2∆/leu2 lys2-128∆/ lys2-128Δ his3∆200/his3 trp1∆63/trp1 CEN1::URA3/CEN1 CAN1/can1-100* pJD190 (*HIS3*-containing centromeric plasmid with *hta1-S122A-HTB1)*.

**MD805:** *MAT***a/***MAT*α *(hta1-htb1)∆::LEU2/hta1-htb1)∆::LEU2 (hta2-htb2)∆::TRP1/ (hta2-htb2)∆::TRP1 ura3-52/ura3 leu2∆/leu2 lys2-128∆/ lys2-128Δ his3∆200/his3 trp1∆63/trp1 CAN1/can1-100 pAB6 (HTA1-HTB1; URA3)* pJD151 (*HIS3*-containing centromeric plasmid with *hta1-S129A-HTB1)*.

**MD806.** *MAT***a/***MAT*α *(hta1-htb1)∆::LEU2/hta1-htb1)∆::LEU2 (hta2-htb2)∆::TRP1/ (hta2-htb2)∆::TRP1 ura3-52/ura3 leu2∆/leu2 lys2-128∆/ lys2-128Δ his3∆200/his3 trp1∆63/trp1 CAN1/can1-100)* pJD151 (*HIS3*-containing centromeric plasmid with *hta1-S129A-HTB1)*.

**MD807:** *MAT***a/***MAT*α *(hta1-htb1)∆::LEU2/hta1-htb1)∆::LEU2 (hta2-htb2)∆::TRP1/ (hta2-htb2)∆::TRP1 ura3-52/ura3 leu2∆/leu2 lys2-128∆/ lys2-128Δ his3∆200/his3 trp1∆63/trp1 CAN1/can1-100 pAB6 (HTA1-HTB1; URA3)* pJD190 (*HIS3*-containing centromeric plasmid with *hta1-S122A-HTB1)*.

**MD808.** *MAT***a/***MAT*α *(hta1-htb1)∆::LEU2/hta1-htb1)∆::LEU2 (hta2-htb2)∆::TRP1/ (hta2-htb2)∆::TRP1 ura3-52/ura3 leu2∆/leu2 lys2-128∆/ lys2-128Δ his3∆200/his3 trp1∆63/trp1 CAN1/can1-100* pJD190 (*HIS3*-containing centromeric plasmid with *hta1-S122A-HTB1)*.

**SGK177.** *MAT***a/***MAT*α *(hta1-htb1)∆::LEU2/hta1-htb1)∆::LEU2 (hta2-htb2)∆::TRP1/ (hta2-htb2)∆::TRP1 ura3-52/ura3 leu2∆/leu2 lys2-128∆/ lys2-128Δ his3∆200/his3 CEN1::URA3/CEN1 trp1∆63/trp1 CAN1/can1-100* pJD190 (*HIS3*-containing centromeric plasmid with *hta1-S122A-HTB1)*.

**MD809:** *MAT***a/***MAT*α *(hta1-htb1)∆::LEU2/hta1-htb1)∆::LEU2 (hta2-htb2)∆::TRP1/ (hta2-htb2)∆::TRP1 ura3-52/ura3 leu2∆/leu2 lys2-128∆/ lys2-128Δ his3∆200/his3 trp1∆63/trp1 CAN1/can1-100* pAB6 *(HTA1-HTB1; URA3)* pJD197 (*HIS3*-containing centromeric plasmid with *hta1-S121A, S128A-HTB1)*.

**MD810.** *MAT***a/***MAT*α *(hta1-htb1)∆::LEU2/hta1-htb1)∆::LEU2 (hta2-htb2)∆::TRP1/ (hta2-htb2)∆::TRP1 ura3-52/ura3 leu2∆1/leu2 lys2-128∆/ lys2-128Δ his3∆200/his3 trp1∆63/trp1 CAN1/can1-100* pJD197 (*HIS3*-containing centromeric plasmid with *hta1-S121A, S128A-HTB1)*.

**MD814.** *MAT***a/***MAT*α *HTA1 HTA2 /hta1-htb1)∆::LEU2 HTA2 HTB2/ (hta2-htb2)∆::TRP1 ura3-52/ura3 leu2∆1/leu2 lys2-128∆/ lys2-128Δ his3∆200/his3 trp1∆63/trp1 CAN1/can1-100* pAB6*(HTA1-HTB1; URA3) bub1Δ::Hyg/bub1Δ::Hyg.*

**MD815.** *MAT***a/***MAT*α *HTA1 HTA2 /hta1-htb1)∆::LEU2 HTA2 HTB2/ (hta2-htb2)∆::TRP1 ura3-52/ura3 leu2∆1/leu2 lys2-128∆/ lys2-128Δ his3∆200/his3 trp1∆63/trp1 CAN1/can1-100* pAB6*(HTA1-HTB1; URA3) bub1Δ::Hyg/bub1Δ::Hyg.*

**MD816.** *MAT***a/***MAT*α *HTA1 HTA2 /hta1-htb1)∆::LEU2 HTA2 HTB2/ (hta2-htb2)∆::TRP1 ura3-52/ura3 leu2∆1/leu2 lys2-128∆/ lys2-128Δ his3∆200/his3 trp1∆63/trp1 CAN1/can1-100 bub1Δ::Hyg/bub1Δ::Hyg.*

**MD817.** *MAT***a/***MAT*α *HTA1 HTA2 /hta1-htb1)∆::LEU2 HTA2 HTB2/ (hta2-htb2)∆::TRP1 ura3-52/ura3 leu2∆1/leu2 lys2-128∆/ lys2-128Δ his3∆200/his3 trp1∆63/trp1 CAN1/can1-100 bub1Δ::Hyg/bub1Δ::Hyg.*

**MD836-1.** *MAT***a/***MAT*α *(hta1-htb1)∆::LEU2/hta1-htb1)∆::LEU2 (hta2-htb2)∆::TRP1/ (hta2-htb2)∆::TRP1 ura3-52/ura3 leu2∆1/leu2 lys2-128∆/ lys2-128Δ his3∆200/his3 trp1∆63/trp1 CAN1/can1-100 CEN1::URA3/CEN1* pJD150-Kan *(HTA1-HTB1; KanMX).*

**MD913.** *MAT***a/***MAT*α *(hta1-htb1)∆::LEU2/hta1-htb1)∆::LEU2 (hta2-htb2)∆::TRP1/ (hta2-htb2)∆::TRP1 ura3-52/ura3 leu2∆1/leu2 lys2-128∆/ lys2-128Δ his3∆200/his3 trp1∆63/trp1 CAN1/can1-100 CEN1::URA3/CEN1* pJD151-HIS3 *(hta1-S129A-HTB1; HIS3).*

**MD914.** *MAT***a/***MAT*α *(hta1-htb1)∆::LEU2/hta1-htb1)∆::LEU2 (hta2-htb2)∆::TRP1/ (hta2-htb2)∆::TRP1 ura3-52/ura3 leu2∆1/leu2 lys2-128∆/ lys2-128Δ his3∆200/his3 trp1∆63/trp1 CAN1/can1-100 CEN1::URA3/CEN1* pJD197-HIS3 (*hta1-S122A hta1-S129A*-HIS3).

**MD838-1.** *MAT***a/***MAT*α *(hta1-htb1)∆::LEU2/hta1-htb1)∆::LEU2 (hta2-htb2)∆::TRP1/ (hta2-htb2)∆::TRP1 ura3-52/ura3 leu2∆1/leu2 lys2-128∆/ lys2-128Δ his3∆200/his3 trp1∆63/trp1 CAN1/can1-100 CEN1::URA3/CEN1* pJD150-Kan *(HTA1-HTB1; KanMX)* pJD150-HIS3 (*HTA1-HTB1; HIS3).*

**MD842-1,2.** *MAT***a/***MAT*α *(hta1-htb1)∆::LEU2/hta1-htb1)∆::LEU2 (hta2-htb2)∆::TRP1/ (hta2-htb2)∆::TRP1 ura3-52/ura3 leu2∆1/leu2 lys2-128∆/ lys2-128Δ his3∆200/his3 trp1∆63/trp1 CAN1/can1-100 CEN1::URA3/CEN1* pJD150-HIS3 (*HTA1-HTB1; HIS3).*

**MD840-1.** *MAT***a/***MAT*α *(hta1-htb1)∆::LEU2/hta1-htb1)∆::LEU2 (hta2-htb2)∆::TRP1/ (hta2-htb2)∆::TRP1 ura3-52/ura3 leu2∆1/leu2 lys2-128∆/ lys2-128Δ his3∆200/his3 trp1∆63/trp1 CAN1/can1-100 CEN1::URA3/CEN1* pJD150-Kan *(HTA1-HTB1; KanMX)* pJD190 *(hta1-S121A-HTB1; HIS3).*

**MD844-1.** *MAT***a/***MAT*α *(hta1-htb1)∆::LEU2/hta1-htb1)∆::LEU2 (hta2-htb2)∆::TRP1/ (hta2-htb2)∆::TRP1 ura3-52/ura3 leu2∆1/leu2 lys2-128∆/ lys2-128Δ his3∆200/his3 trp1∆63/trp1 CAN1/can1-100 CEN1::URA3/CEN1* pJD190 *(hta1-S121A-HTB1; HIS3).*

**MD837-1.** *MAT***a/***MAT*α *(hta1-htb1)∆::LEU2/hta1-htb1)∆::LEU2 (hta2-htb2)∆::TRP1/ (hta2-htb2)∆::TRP1 ura3-52/ura3 leu2∆1/leu2 lys2-128∆/ lys2-128Δ his3∆200/his3 trp1∆63/trp1 CAN1/can1-100 CEN1::URA3/CEN1* pJD150-Kan (*HTA1-HTB1; KanMX).*

**MD841-1.** *MAT***a/***MAT*α *(hta1-htb1)∆::LEU2/hta1-htb1)∆::LEU2 (hta2-htb2)∆::TRP1/ (hta2-htb2)∆::TRP1 ura3-52/ura3 leu2∆1/leu2 lys2-128∆/ lys2-128Δ his3∆200/his3 trp1∆63/trp1 CAN1/can1-100 CEN1::URA3/CEN1* pJD150-Kan (*HTA1-HTB1; KanMX)* pJD190 *(hta1-S121A-HTB1; HIS3)*.

**MD845-1.** *MAT***a/***MAT*α *(hta1-htb1)∆::LEU2/hta1-htb1)∆::LEU2 (hta2-htb2)∆::TRP1/ (hta2-htb2)∆::TRP1 ura3-52/ura3 leu2∆1/leu2 lys2-128∆/ lys2-128Δ his3∆200/his3 trp1∆63/trp1 CAN1/can1-100 CEN1::URA3/CEN1* pJD190 *(hta1-S121A-HTB1; HIS3)*.

**MD831.** *MAT***a/***MAT*α *(hta1-htb1)∆::LEU2/hta1-htb1)∆::LEU2 (hta2-htb2)∆::TRP1/ (hta2-htb2)∆::TRP1 ura3-52/ura3 leu2∆1/leu2 lys2-128∆/ lys2-128Δ his3∆200/his3 trp1∆63/trp1 CAN1/can1-100 CEN1::URA3/CEN1 sml1∆::Hyg/sml1∆::Hyg* pJD150-Kan *(HTA1-HTB1; KanMX).*

**MD850-1.** *MAT***a**/*MATα* *(hta1-htb1)∆::LEU2/hta1-htb1)∆::LEU2 (hta2-htb2)∆::TRP1/ (hta2-htb2)∆::TRP1 ura3-52/ura3 leu2∆1/leu2 lys2-128∆/ lys2-128Δ his3∆200/his3 trp1∆63/trp1 CAN1/can1-100 CEN1::URA3/CEN1 sml1∆::Hyg/sml1∆::Hyg* pJD150-HIS3 [*HTA1-HTB1; HIS3].*

**MD851-1.** *MAT***a/***MAT*α *(hta1-htb1)∆::LEU2/hta1-htb1)∆::LEU2 (hta2-htb2)∆::TRP1/ (hta2-htb2)∆::TRP1 ura3-52/ura3 leu2∆1/leu2 lys2-128∆/ lys2-128Δ his3∆200/his3 trp1∆63/trp1 CAN1/can1-100 CEN1::URA3/CEN1 sml1∆::Hyg/sml1∆::Hyg* pJD190*(hta1-S121A-HTB1; HIS3).*

**MD852-1,2:** MAT**a**/*MATα* *(hta1-htb1)∆::LEU2/hta1-htb1)∆::LEU2 (hta2-htb2)∆::TRP1/ (hta2-htb2)∆::TRP1 ura3-52/ura3 leu2∆1/leu2 lys2-128∆/ lys2-128Δ his3∆200/his3 trp1∆63/trp1 CAN1/can1-100 CEN1::URA3/CEN1 sml1∆::Hyg/sml1∆::Hyg* pJD150-HIS3 [*HTA1-HTB1; HIS3].*

**MD853-1,2.** *MAT***a**/*MATα* *(hta1-htb1)∆::LEU2/hta1-htb1)∆::LEU2 (hta2-htb2)∆::TRP1/(hta2-htb2)∆::TRP1 ura3-52/ura3 leu2∆1/leu2 lys2-128∆/ lys2-128Δ his3∆200/his3 trp1∆63/trp1 CAN1/can1-100 CEN1::URA3/CEN1 sml1∆::Hyg/sml1∆::Hyg* pJD190*(hta1-S121A-HTB1; HIS3)*.

**MD832.** *MAT***a/***MAT*α *(hta1-htb1)∆::LEU2/hta1-htb1)∆::LEU2 (hta2-htb2)∆::TRP1/(hta2-htb2)∆::TRP1 ura3-52/ura3 leu2∆1/leu2 lys2-128∆/ lys2-128Δ his3∆200/his3 trp1∆63/trp1 CAN1/can1-100 CEN1::URA3/CEN1 sml1∆::Hyg/sml1∆::Hyg* pJD150-Kan *(HTA1-HTB1; KanMX).*

**MD861-1,2.** *MAT***a**/*MATα* *(hta1-htb1)∆::LEU2/hta1-htb1)∆::LEU2 (hta2-htb2)∆::TRP1/(hta2-htb2)∆::TRP1 ura3-52/ura3 leu2∆1/leu2 lys2-128∆/ lys2-128Δ his3∆200/his3 trp1∆63/trp1 CAN1/can1-100 CEN1::URA3/CEN1 bub1Δ::Hyg/bub1Δ::Hyg* p*MEC1-BUB1-LYS2* pJD150-Kan *(HTA1-HTB1; KanMX).* Same genotype as MD862-1,2.

**SGK659.** *MAT***a**/*MATα* *(hta1-htb1)∆::LEU2/hta1-htb1)∆::LEU2 (hta2-htb2)∆::TRP1/(hta2-htb2)∆::TRP1 ura3-52/ura3 leu2∆1/leu2 lys2-128∆/ lys2-128Δ his3∆200/his3 trp1∆63/trp1 CAN1/can1-100 CEN1::URA3/CEN1 bub1Δ::Hyg/bub1Δ::Hyg* pJD190 *(hta1-S122A-HTB1; HIS3).* Same genotype as SGK663.

**MD862-1,2**. *MAT***a**/*MATα* *(hta1-htb1)∆::LEU2/hta1-htb1)∆::LEU2 (hta2-htb2)∆::TRP1/(hta2-htb2)∆::TRP1 ura3-52/ura3 leu2∆1/leu2 lys2-128∆/ lys2-128Δ his3∆200/his3 trp1∆63/trp1 CAN1/can1-100 CEN1::URA3/CEN1 bub1Δ::Hyg/bub1Δ::Hyg* p*MEC1-BUB1-LYS2* pJD150-Kan *(HTA1-HTB1; KanMX).* Same genotype as MD861-1,2.

**SGK663.** *MAT***a**/*MATα* *(hta1-htb1)∆::LEU2/hta1-htb1)∆::LEU2 (hta2-htb2)∆::TRP1/(hta2-htb2)∆::TRP1 ura3-52/ura3 leu2∆1/leu2 lys2-128∆/ lys2-128Δ his3∆200/his3 trp1∆63/trp1 CAN1/can1-100 CEN1::URA3/CEN1 bub1Δ::Hyg/bub1Δ::Hyg* pJD190 *(hta1-S122A-HTB1; HIS3).* Same genotype as SGK659.

**MD865-1,2.** *MAT***a/***MATα* *(hta1-htb1)∆::LEU2/hta1-htb1)∆::LEU2 (hta2-htb2)∆::TRP1/(hta2-htb2)∆::TRP1 ura3-52/ura3 leu2∆1/leu2 lys2-128∆/ lys2-128Δ his3∆200/his3 trp1∆63/trp1 CAN1/can1-100 CEN1::URA3/CEN1* pJD150-HIS3 [*HTA1-HTB1; HIS3]*  *bub1Δ::Hyg/bub1Δ::Hyg.*

**MD868-1,2.** *MAT***a**/*MATα* *(hta1-htb1)∆::LEU2/hta1-htb1)∆::LEU2 (hta2-htb2)∆::TRP1/(hta2-htb2)∆::TRP1 ura3-52/ura3 leu2∆1/leu2 lys2-128∆/ lys2-128Δ his3∆200/his3 trp1∆63/trp1 CAN1/can1-100 CEN1::URA3/CEN1* pJD150-HIS3 [*HTA1-HTB1; HIS3]*  *bub1Δ::Hyg/bub1Δ::Hyg.* Same genotype as MD865-1,2.

**MD876-1,2**. *MAT***a**/*MATα* *(hta1-htb1)∆::LEU2/hta1-htb1)∆::LEU2 (hta2-htb2)∆::TRP1/(hta2-htb2)∆::TRP1 ura3-52/ura3 leu2∆1/leu2 lys2-128∆/ lys2-128Δ his3∆200/his3 trp1∆63/trp1 CEN1::URA3/CEN1 sml1Δ::loxP/sml1Δ::loxP* pJD150-Kan *[HTA1-HTB1; KanMX) tel1Δ::loxP/tel1Δ::loxP* p*MEC1-BUB1-LYS2 mec1Δ::Nat/mec1Δ::Nat*

**MD877-1,2**. *MAT***a**/*MATα* *(hta1-htb1)∆::LEU2/hta1-htb1)∆::LEU2 (hta2-htb2)∆::TRP1/(hta2-htb2)∆::TRP1 ura3-52/ura3 leu2∆1/leu2 lys2-128∆/ lys2-128Δ his3∆200/his3 trp1∆63/trp1 CAN1/can1-100 CEN1::URA3/CEN1 sml1Δ::loxP/sml1Δ::loxP* pJD150-Kan *[HTA1-HTB1; KanMX) tel1Δ::loxP/tel1Δ::loxP* p*MEC1-BUB1-LYS2 mec1Δ::Nat/mec1Δ::Nat*

**MD915.** *MAT***a**/*MATα* *(hta1-htb1)∆::LEU2/hta1-htb1)∆::LEU2 (hta2-htb2)∆::TRP1/(hta2-htb2)∆::TRP1 ura3-52/ura3 leu2∆1/leu2 lys2-128∆/ lys2-128Δ his3∆200/his3 trp1∆63/trp1 CAN1/can1-100 CEN1::URA3/CEN1 sml1Δ::Hyg/sml1Δ::Hyg* pJD150-HIS3 *[HTA1-HTB1; HIS3) tel1Δ::loxP/tel1Δ::loxP mec1Δ::Nat/mec1Δ::Nat*

**MD917.** *MAT***a**/*MATα* *(hta1-htb1)∆::LEU2/hta1-htb1)∆::LEU2 (hta2-htb2)∆::TRP1/(hta2-htb2)∆::TRP1 ura3-52/ura3 leu2∆1/leu2 lys2-128∆/ lys2-128Δ his3∆200/his3 trp1∆63/trp1 CAN1/can1-100 CEN1::URA3/CEN1 sml1Δ::Hyg/sml1Δ::Hyg* *tel1Δ::loxP/tel1Δ::loxP mec1Δ::Nat/mec1Δ::Nat* pJD190*(hta1-S121A-HTB1; HIS3)*.

**MD918.** *MAT***a**/*MATα* *(hta1-htb1)∆::LEU2/hta1-htb1)∆::LEU2 (hta2-htb2)∆::TRP1/(hta2-htb2)∆::TRP1 ura3-52/ura3 leu2∆1/leu2 lys2-128∆/ lys2-128Δ his3∆200/his3 trp1∆63/trp1 CAN1/can1-100 CEN1::URA3/CEN1 sml1Δ::Hyg/sml1Δ::Hyg* *tel1Δ::loxP/tel1Δ::loxP mec1Δ::Nat/mec1Δ::Nat* pJD190*(hta1-S121A-HTB1; HIS3)*.

**MD880-1**. *MAT***a**/*MATα* *(hta1-htb1)∆::LEU2/hta1-htb1)∆::LEU2 (hta2-htb2)∆::TRP1/(hta2-htb2)∆::TRP1 ura3-52/ura3 leu2∆1/leu2 lys2-128∆/ lys2-128Δ his3∆200/his3 trp1∆63/trp1 CAN1/can1-100 CEN1::URA3/CEN1 sml1Δ::loxP/sml1Δ::loxP* *tel1Δ::loxP/tel1Δ::loxP mec1Δ::Nat/mec1Δ::Nat* pJD150-Kan *[HTA1-HTB1; KanMX)* p*MEC1-BUB1-LYS2 bub1Δ::Hyg/bub1Δ::Hyg*

**MD881-1**. *MAT***a**/*MATα* *(hta1-htb1)∆::LEU2/hta1-htb1)∆::LEU2 (hta2-htb2)∆::TRP1/(hta2-htb2)∆::TRP1 ura3-52/ura3 leu2∆1/leu2 lys2-128∆/ lys2-128Δ his3∆200/his3 trp1∆63/trp1 CAN1/can1-100 CEN1::URA3/CEN1 sml1Δ::loxP/sml1Δ::loxP* *tel1Δ::loxP/tel1Δ::loxP mec1Δ::Nat/mec1Δ::Nat* pJD150-Kan *[HTA1-HTB1; KanMX)* p*MEC1-BUB1-LYS2 bub1Δ::Hyg/bub1Δ::Hyg*

**MD882-2/MD883-1.** *MAT***a**/*MATα* *(hta1-htb1)∆::LEU2/hta1-htb1)∆::LEU2 (hta2-htb2)∆::TRP1/(hta2-htb2)∆::TRP1 ura3-52/ura3 leu2∆1/leu2 lys2-128∆/ lys2-128Δ his3∆200/his3 trp1∆63/trp1 CAN1/can1-100 CEN1::URA3/CEN1 sml1Δ::Hyg/sml1Δ::Hyg* *tel1Δ::loxP/tel1Δ::loxP mec1Δ::Nat/mec1Δ::Nat* pJD150-HIS3 *[HTA1-HTB1; HIS3)* *bub1Δ::Hyg/bub1Δ::Hyg*

**MD884-1/MD885-1.** *MAT***a/***MATα* *(hta1-htb1)∆::LEU2/hta1-htb1)∆::LEU2 (hta2-htb2)∆::TRP1/(hta2-htb2)∆::TRP1 ura3-52/ura3 leu2∆1/leu2 lys2-128∆/ lys2-128Δ his3∆200/his3 trp1∆63/trp1 CAN1/can1-100 CEN1::URA3/CEN1 sml1Δ::Hyg/sml1Δ::Hyg* *tel1Δ::loxP/tel1Δ::loxP mec1Δ::Nat/mec1Δ::Nat* pJD190-HIS3 *[hta1-S122A; HIS3)* *bub1Δ::Hyg/bub1Δ::Hyg.*

**MD1002.** *MAT***a/***MAT*α *(hta1-htb1)∆::LEU2/hta1-htb1)∆::LEU2 (hta2-htb2)∆::TRP1/ (hta2-htb2)∆::TRP1 ura3-52/ura3 leu2∆1/leu2 lys2-128∆/ lys2-128Δ his3∆200/his3 trp1∆63/trp1 CAN1/can1-100 CEN1::URA3/CEN1 I-9500::Hyg/I-9500* pJD150-Kan *(hta1-S121A-HTB1;KanMX).*

**MD1003.** *MAT***a/***MAT*α *(hta1-htb1)∆::LEU2/hta1-htb1)∆::LEU2 (hta2-htb2)∆::TRP1/ (hta2-htb2)∆::TRP1 ura3-52/ura3 leu2∆1/leu2 lys2-128∆/ lys2-128Δ his3∆200/his3 trp1∆63/trp1 CAN1/can1-100 sml1Δ::loxP/sml1Δ::loxP* *tel1Δ::loxP/tel1Δ::loxP mec1Δ::Nat/mec1Δ::Nat CEN1::URA3/CEN1 I-9500::Hyg/I-9500* pJD150-Kan *(hta1-S121A-HTB1;KanMX)* p*MEC1-BUB1-LYS2.*

**MD1004.** *MAT***a/***MAT*α *(hta1-htb1)∆::LEU2/hta1-htb1)∆::LEU2 (hta2-htb2)∆::TRP1/ (hta2-htb2)∆::TRP1 ura3-52/ura3 leu2∆1/leu2 lys2-128∆/ lys2-128Δ his3∆200/his3 trp1∆63/trp1 CAN1/can1-100 CEN1::URA3/CEN1 I-9500::Hyg/I-9500* pJD190-HIS3 *(hta1-S121A-HTB1; HIS3).*

**MD1005.** *MAT***a/***MAT*α *(hta1-htb1)∆::LEU2/hta1-htb1)∆::LEU2 (hta2-htb2)∆::TRP1/ (hta2-htb2)∆::TRP1 ura3-52/ura3 leu2∆1/leu2 lys2-128∆/ lys2-128Δ his3∆200/his3 trp1∆63/trp1 CAN1/can1-100 sml1Δ::loxP/sml1Δ::loxP* *tel1Δ::loxP/tel1Δ::loxP mec1Δ::Nat/mec1Δ::Nat CEN1::URA3/CEN1 I-9500::Hyg/I-9500* pJD190-HIS3 *(hta1-S121A-HTB1; HIS3).*
