# Supplementary material for: A tale of two serines: the effects of histone H2A mutations S122A and S129A on chromosome nondisjunction in Saccharomyces cerevisiae
Source: Genetics. 2024 Nov 18;229(1):iyae194. doi: 10.1093/genetics/iyae194 (PMC11708911; doi:10.1093/genetics/iyae194)
Supplement: iyae194_Supplementary_Data [file iyae194_supplementary_data.zip › Supplemental_Material_and_Methods_GENETICS-2024-307465.docx]

**Supplemental Materials and Methods**

For strains used to analyze unselected aneuploidy (MD802, MD806, MD808, MD810, MD816/MD817), two types of constructions were used. For those diploids in which FY406 was in the pedigree for the *MAT***a** strains (MD802, MD806, MD808, and MD810), the diploids were homozygous for deletions of the histone genes on the chromosome. For the MD816 and MD817 strains (*bub1/bub1*), as well as the progenitor strains MD814 and MD815, the diploids were heterozygous for the histone deletions on the chromosome.

In the list of strains below, if two strain names are listed for the same genotype, these strains are isogenic but represent independent transformants or independent diploids made in the same cross.

**Details of strain constructions**

As described in the main text, most of the diploids used in the study were the result of crosses of haploids isogenic (except for changes introduced by transformation) to FY406 (derivative of S288c; Hirschhorn *et al.*, 1995) with haploids isogenic with MD761-5-16A. The genotype of FY406 is: *MAT***a** *(hta1-htb1)∆::LEU2 (hta2-htb2)∆::TRP1 ura3-52 leu2∆1 lys2-128∆ his3∆200 trp1∆63 pAB6 (HTA1-HTB1; URA3)*. The plasmid pAB6 contains the wild-type *HTA1* and *HTB1* genes in the centromere-containing vector pRS316 (Hirschhorn *et al.*, 1995).

The strain MD761-5-16A has the genotype: *MAT*α *(hta1-htb1)∆::LEU2 (hta2-htb2)∆::TRP1 leu2 his3 ura3 lys2-128Δ trp1 can1-100 pAB6 (HTA1-HTB1; URA3)*. This strain was a spore derived from MD761-5, a diploid generated by a cross of FY406 and RCY278-5A. RCY278-5A has the genotype: *MATα leu2-3,112 his3-11,15 ura3-1 ade2-1 trp1-1 can1-100 RAD5.* RCY278-5A was a spore derived from the diploid RCY278 (isogenic with W303-1A; Craven and Petes, 2001).

The construction of haploid and diploid strains used in our study are described below.

**Construction and genotypes of *MAT*a derivatives used in the study**

**S288c:** Wild-type *MAT***a** haploid (<https://www.yeastgenome.org/strain/s288c>),

**SGK42** (derivative of S288c): S288c was transformed with a PCR fragment produced by PCR amplification of the plasmid pAG32 (Goldstein and McCu178er, 1999) with the primers LYS2-kanF (5'- GGCATCGCACAGTTTTAGCGAGGAAAACTCTTCAATAGTTTTGCCAGCGGCAGCTGAAGCTTCGTACGC) and LYS2-kanR (5'- CCTTA GCAGACTAACGCCAGCTGATTTACAGTTCTTATTCAATAACTAAAGGCCACTAGTGGATCTG). *MAT***a** *lys2*Δ::*Hyg.*

**SGK80** (derivative of S288c): Transformant of SGK42 with PCR fragment generated by amplification of the plasmid pFA6a-*kanMX4* with primers ura3reg-kanF(5'-ATTTATGGTGAAGGATAAGTTTTGACCATCAAAGAAGGTTAATGTGGCTGCAGCTGAAGCTTCGTACGC) and ura3reg-kanR (5'-AGCTTTTTCTTTCCAATTTTTTTTTTTTCG TCATTATAGAAATCATTACGACCGAGATTCCAGGCCACTAGTGGATCTG). *MAT***a** *lys2*Δ::*Hyg ura3*Δ::*Kan*

**FY602** (derivative of S288c, provided by Fred Winston, Harvard Medical School; https://winston-strain-finder.hms.harvard.edu/yeast/12493**):** *MAT***a** *ura3-52 leu2-∆1 lys2-128∆ his3-∆200 trp1-∆63*

**MD813:** Deletion of *BUB1* in FY602 using a PCR fragment obtained by amplifying the plasmid pAG32 (Goldstein and McCusker, 1999) with the primers Bub1-KO-F (5’-CATGGAAAGATTATTGACGGTTCCTATTCTTTGAATGTTAACGCTGACCAGGAACGTACGCTGCAGGTCGAC) and Bub1-KO-R (5’-CTGGCAGGACACCAAAAAGTCACCTATGCGGGAGATGAAGGCATATTTATTCAATCGATGAATTCGAGCTCG). *MAT***a** *ura3-52 leu2∆1 lys2-128∆ his3∆200 trp1∆63 bub1∆::Hyg.*

**FY406** (derivative of S288c): *MAT***a** *(hta1-htb1)∆::LEU2 (hta2-htb2)∆::TRP1 ura3-52 leu2∆1 lys2-128∆ his3∆200 trp1∆63* pAB6 *(HTA1-HTB1; URA3)*; pAB6 contains the wild-type *HTA1* and *HTB1* genes in the centromere-containing vector pRS316 (Hirschhorn *et al.*, 1995).

**FY406-pJD150-HIS3:** Transformant of FY406 with the plasmid pJD150 (*HIS3*-containing centromeric plasmid with *HTA1-HTB1*; Harvey *et al.*, 2005). *MAT***a** *(hta1-htb1)∆::LEU2 (hta2-htb2)∆::TRP1 ura3-52 leu2∆1 lys2-128∆ his3∆200 trp1∆63* pAB6 *(HTA1-HTB1; URA3)* pJD150-HIS3 *(HTA1-HTB1; HIS3 )*.

**SGK330/SGK331:** Derivative of FY406-pJD150-HIS3 that lacks the pAB6 plasmid. *MAT***a** *(hta1-htb1)∆::LEU2 (hta2-htb2)∆::TRP1 ura3-52 leu2∆1 lys2-128∆ his3∆200 trp1∆63* pJD150-HIS3 *(HTA1-HTB1; HIS3 )*.

**SGK334/SGK336:** Derivative of SGK330/SGK331 that contains a *URA3* gene integrated near *CEN1*. This construction was generated by transformation using a PCR product created by amplification of genomic DNA of S288c with the primers URA3-CEN1-F (5'-CTCAATCTTGAGTCGTCCATGTATCGTTTTATAATACTTTTTTAAGCACTGGTTCAGGGTCCATAAAG) and URA3-CEN1-R (5'-CTGAAAGTTCATCTCATTTCAGTAACAGTACTTCAATGGAATATTTATTAAG AAACATGCATTTACTTATAATACAG). The resulting PCR fragment containing the wild-type *URA3* gene was integrated into chromosome I between positions 151,749 and 151,750. *MAT***a** *(hta1-htb1)∆::LEU2 (hta2-htb2)∆::TRP1 ura3-52 leu2∆1 lys2-128∆ his3∆200 trp1∆63* pJD150-HIS3 *(HTA1-HTB1; HIS3) CEN1::URA3.*

**MD825-1,2/MD826-1,2:** Derivative of SGK334/SGK336 with *sml1∆::loxP-Hyg-loxP* deletion. The progenitor haploid was transformed with a PCR fragment containing the *Hyg* gene flanked by *loxP* sites generated with the primers sml1-kanF (5'-GCTCCTTTGTGATCTTACGGCTCACTAACCTCTCTTCAACTGCTCAATAATTTCCCGCTCAGCTGAAGCTTCGTACGC) and sml1-kanR (5'-AAGAAAAGAACAGAACTAGTGGGAAATGGAAAGAGAAAAGAAAAGAGTATGAAAGGAACTAGGCCACTAGTGGATCTG) using the plasmid pSR955 (Guo et al., 2017) as a template. *MAT***a** *(hta1-htb1)∆::LEU2 (hta2-htb2)∆::TRP1 ura3-52 leu2∆1 lys2-128∆ his3∆200 trp1∆63* pJD150-HIS3 *(HTA1-HTB1; HIS3) CEN1::URA3 sml1∆::loxP-Hyg-loxP.*

**MD863:** Derivative of MD825-1,2/MD826-1,2 with substitution of *sml1∆::loxP-Hyg-loxP* with *sml1∆::loxP-Nat-loxP.* The strain was generated by transformation with a PCR fragment produced by amplification of the pAG25 plasmid (Goldstein and McCusker, 1999) with the primers Ptef-F (5'-GACATGGAGGCCCAGAATAC) and Ttef-R (5'-CAGTATAGCGACCAGCATTC). *MAT***a** *(hta1-htb1)∆::LEU2 (hta2-htb2)∆::TRP1 ura3-52 leu2∆1 lys2-128∆ his3∆200 trp1∆63* pJD150-HIS3 *(HTA1-HTB1; HIS3) CEN1::URA3 sml1∆::loxP-Nat-loxP.*

**MD829-1/MD830-1:** Transformant of MD825-1,2/MD826-1,2 with pJD150-Kan and loss of pJD150-HIS3. *MAT***a** *(hta1-htb1)∆::LEU2 (hta2-htb2)∆::TRP1 ura3-52 leu2∆1 lys2-128∆ his3∆200 trp1∆63 CEN1::URA3 sml1Δ::loxP-Hyg-loxP)* pJD150-Kan *(HTA1-HTB1; KanMX).* The pJD150-Kan plasmid was constructed using a PCR product containing the Kan gene by PCR amplification of the vector pFA6a-kanMX (Wach *et al.*, 1994) with the primers pJD150-his-kan-F (5’ CTGGCTTAACTATGCGGCATCAGAGCAGATTGTACTGAGAGTGCACCATACAGCTGAAGCTTCGTACGC) and pJD150-his-kan-R (5’ GGTATTTTCTCCTTACGCAT TGTGCGGTATTTCACACCGCATAGATCCGAGGCCACTAGTGGATCTG). This product was used to transform the strain MD802, which contained pDJ150-HIS3, and strains were selected for resistance to geneticin and screened for the His^-^ phenotype. The resulting plasmid was transformed into *E. coli*, and used for subsequent yeast transformation experiments.

**TDP4:** Derivative of SGK334/SGK336 with pJD150-Kan plasmid. *MAT***a** *(hta1-htb1)∆::LEU2 (hta2-htb2)∆::TRP1 ura3-52 leu2∆1 lys2-128∆ his3∆200 trp1∆63* pJD150-HIS3 *(HTA1-HTB1; HIS3) CEN1::URA3* pJD150-Kan *(HTA1-HTB1; KanMX)*.

**MD833-1,2/MD834-1,2:** Derivatives of TDP4 that have lost the pJD150-HIS3 plasmid. *MAT***a** *(hta1-htb1)∆::LEU2 (hta2-htb2)∆::TRP1 ura3-52 leu2∆1 lys2-128∆ his3∆200 trp1∆63 CEN1::URA3* pJD150-Kan *(HTA1-HTB1; KanMX)*.

**MD1000-1:** Derivative of MD833-1 with a *Hyg* insertion on chromosome I between coordinates 9500 and 9501. This strain was constructed by transformation of MD833-1 with a PCR fragment generated by amplification of plasmid pAG32 DNA (Goldstein and McCusker, 1999) with the primers I-9.8KB-kanF (5’TGTTAAAATACAGGGGTAAGACATTGGTGGATATTCAACAAGATCCGATAcagctgaagcttcgtacgc) and I-9.8KB-kanR (5’TGCATCGCAGTAATATGTAGAGCACAATTTGTAGAAATCGGAATTGGAGGaggccactagtggatctg). *MAT***a** *(hta1-htb1)∆::LEU2 (hta2-htb2)∆::TRP1 ura3-52 leu2∆1 lys2-128∆ his3∆200 trp1∆63 CEN1::URA3 I-9500::Hyg* pJD150-Kan *(HTA1-HTB1; KanMX)*.

**MD1001-1:** Derivative of MD875-1 with a *Hyg* insertion on chromosome I between coordinates 9500 and 9501. This strain was constructed by transformation of MD875-1 with a PCR fragment generated by amplification of plasmid pAG32 DNA (Goldstein and McCusker, 1999) with the primers I-9.8KB-kanF (5’TGTTAAAATACAGGGGTAAGACATTGGTGGATATTCAACAAGATCCGATAcagctgaagcttcgtacgc) and I-9.8KB-kanR (5’TGCATCGCAGTAATATGTAGAGCACAATTTGTAGAAATCGGAATTGGAGGaggccactagtggatctg). *MAT***a** *(hta1-htb1)∆::LEU2 (hta2-htb2)∆::TRP1 ura3-52 leu2∆1 lys2-128∆ his3∆200 trp1∆63 CEN1::URA3 I-9500::Hyg sml1Δ::loxP* pJD150-Kan *[HTA1-HTB1; KanMX) tel1Δ::loxP* p*MEC1-BUB1-LYS2 mec1Δ::Nat*

**MD858-1,2/MD859-1,2:** Transformation of MD833-1/MD834-1 with pMEC1-BUB1-LYS2, and a PCR fragment containing the *bub1Δ::Hyg* sequence. The pMEC1-BUB1-LYS2 plasmid was constructed as follows: 1. The *TRP1* gene on the pSAD3-3b plasmid (Desany *et al.,* 1998) was replaced with *LYS2* by transforming the yeast strain YYy16.1-1 diploid containing pSAD3-3B (Yi Yan, UCLA) with a PCR fragment with the *LYS2* gene obtained by amplification of S288c genomic DNA with the primers pRS414-TRP1-LYS2-F (5'-GGTGTCGGG GCTGGCTTAACTATGCGGCATCAGAGCAGATTGTACTGAGAGTGCACCATAAATTCCACTTGCAATTACAT) and pRS414-TRP1-LYS2-R (5'-TTACAATTTCCTGATGCGGTATTTCTCCTTACGCATCGTGCGGTATTTCACACCGCATAAGAACTA GT CTTTCTCC). The resulting plasmid (pSAD3-3B-LYS2) was purified from yeast and used to transform *E. coli*. The plasmid was then linearized with *Pci*I and a PCR product containing the *BUB1* gene was inserted. This fragment was produced by PCR amplification of genomic DNA of S288c with the primers bub1-pci-F (5'-CTACACATGTTATGTTTCATTATTACATTATC) and bub1-pci-R (5'-CTACACATGTTGTCATTGCTATGGAATCTG). The resulting plasmid was pMEC1-BUB1-LYS2. Genotype: *MAT***a** *(hta1-htb1)∆::LEU2 (hta2-htb2)∆::TRP1 ura3-52 leu2∆1 lys2-128∆ his3∆200 trp1∆63 CEN1::URA3* pJD150-Kan *(HTA1-HTB1; KanMX)* pMEC1-BUB1-LYS2 *bub1Δ::Hyg.*

**FY406-pJD151:** Transformant of FY406 with the plasmid pJD151 (*HIS3*-containing centromeric plasmid with *hta1-S129A-HTB1*; Harvey *et al.*, 2005). *MAT***a** *(hta1-htb1)∆::LEU2 (hta2-htb2)∆::TRP1 ura3-52 leu2∆1 lys2-128∆ his3∆200 trp1∆63* pAB6 *(HTA1-HTB1; URA3)* pJD151 *(hta1-S129A-HTB1; HIS3)*.

**FY406-pJD190:** Transformant of FY406 with the plasmid pJD190 (*HIS3*-containing centromeric plasmid with *hta-S122A-HTB1*; Harvey *et al.*, 2005). *MAT***a** *(hta1-htb1)∆::LEU2 (hta2-htb2)∆::TRP1 ura3-52 leu2∆1 lys2-128∆ his3∆200 trp1∆63* pAB6 *(HTA1-HTB1; URA3)* pJD190 *(hta1-S122A-HTB1; HIS3)*.

**FY406-pJD197:** Transformant of FY406 with the plasmid pJD197 (*HIS3*-containing centromeric plasmid with *hta-S122A, S128A-HTB1*; Harvey *et al.*, 2005). *MAT***a** *(hta1-htb1)∆::LEU2 (hta2-htb2)∆::TRP1 ura3-52 leu2∆1 lys2-128∆ his3∆200 trp1∆63* pAB6 *(HTA1-HTB1; URA3)* pJD197 *(hta1-S122A,S128A-HTB1; HIS3)*.

**MD871-1,2:** Transformant of MD829-1/MD830-1 with cassette to replace *TEL1* with the *Nat* gene. The cassette was generated by PCR amplification of genomic DNA of MD863 with the primers tel1-kanF (5'-AAGCAGGAAATTCGAAAAAAAAGCCTTCAAAGAAAAAGGGAAATCAGTGTAACATAGACGCAGCTGAAGCTTCGTACGC) and tel1-kanR (5'-TACATTACTTTTCGTATTTCTATAAACAAAAAAAAGAAGTATAAAGCATCTGCATAGCAAAGGCCACTAGTGGATCTG). *MAT***a** *(hta1-htb1)∆::LEU2 (hta2-htb2)∆::TRP1 ura3-52 leu2∆1 lys2-128∆ his3∆200 trp1∆63 CEN1::URA3 sml1Δ::loxP-Hyg-loxP* pJD150-Kan *[HTA1-HTB1; KanMX] ) tel1Δ::loxP-Nat-loxP*

**MD871-1,2 + pSH47-LYS2(Cre):** Transformant of MD871-1,2 with pSH47-LYS2(Cre) plasmid. The pSH47-LYS2(Cre) plasmid was constructed by replacing the *URA3* gene on pSH47 (Güldener *et al*., 1996) with *LYS2*. This replacement was performed by transformation of the S288c derivative SGK80 (containing pSH47) with a PCR fragment generated by amplifying S288c genomic DNA with the primers pRS414-TRP1-LYS2-F and pRS414-TRP1-LYS2-R (same primer sequences as used for MD858). The plasmid pSH47-LYS2(Cre) was then transformed from yeast into *E. coli*. *MAT***a** *(hta1-htb1)∆::LEU2 (hta2-htb2)∆::TRP1 ura3-52 leu2∆1 lys2-128∆ his3∆200 trp1∆63 CEN1::URA3 sml1Δ::loxP* pJD150-Kan *[HTA1-HTB1; KanMX) tel1Δ::loxP* pSH47-LYS2(Cre).

**MD873-1,2:** Derivative of MD871-1,2 + pSH47-LYS2(Cre) lacking the plasmid pSH47-LYS2(Cre). *MAT***a** *(hta1-htb1)∆::LEU2 (hta2-htb2)∆::TRP1 ura3-52 leu2∆1 lys2-128∆ his3∆200 trp1∆63 CEN1::URA3 sml1Δ::loxP* pJD150-Kan *[HTA1-HTB1; KanMX) tel1Δ::loxP*

**MD875-1,2:** Derivative of MD873-1,2 containing pMEC1-BUB1-LYS2 and *mec1Δ::Nat.* The plasmid pMEC1-BUB1-LYS2 is described above. The *mec1Δ::Nat* construction was made by transforming a PCR fragment obtained using genomic DNA from the strain YYy16.1-1 as a template, and the primers mec1-P604F (5'-GAAGACGAATGGAGAGGCCT) and mec1-T323R (5'-GGGAACCTTACGAGAAGTCAATC). *MAT***a** *(hta1-htb1)∆::LEU2 (hta2-htb2)∆::TRP1 ura3-52 leu2∆1 lys2-128∆ his3∆200 trp1∆63 CEN1::URA3 sml1Δ::loxP* pJD150-Kan *[HTA1-HTB1; KanMX) tel1Δ::loxP* p*MEC1-BUB1-LYS2 mec1Δ::Nat*

**MD879-1,2,3,4:** Transformant of MD875-1 with *bub1Δ::Hyg* cassette. This cassette was generated as described in the construction of MD813*. MAT***a** *(hta1-htb1)∆::LEU2 (hta2-htb2)∆::TRP1 ura3-52 leu2∆1 lys2-128∆ his3∆200 trp1∆63 CEN1::URA3 sml1Δ::loxP* pJD150-Kan *[HTA1-HTB1; KanMX) tel1Δ::loxP* p*MEC1-BUB1-LYS2 mec1Δ::Nat bub1Δ::Hyg.*

**Construction and genotypes of *MATα* derivatives used in the study**

**MD761-5-16A:** *MAT*α *(hta1-htb1)∆::LEU2 (hta2-htb2)∆::TRP1 leu2 his3 ura3 lys2-128Δ trp1 can1-100 pAB6 (HTA1-HTB1; URA3).* Spore of MD761-5.

**MD812-1,2:** Transformation of MD761-5-16A with a PCR fragment containing the *bub1Δ::Hyg* construction (details in the description of MD813). *MAT*α *(hta1-htb1)∆::LEU2 (hta2-htb2)∆::TRP1 leu2 his3 ura3 lys2-128Δ trp1 can1-100* pAB6 *(HTA1-HTB1; URA3) bub1Δ::Hyg.*

**MD835-1,2:** Derivative of MD761-5-16A that lost pAB6 plasmid and gained the pJD150-Kan plasmid. *MAT*α *(hta1-htb1)∆::LEU2 (hta2-htb2)∆::TRP1 leu2 his3 ura3 lys2-128Δ trp1 can1-100* pJD150-Kan *(HTA1-HTB1; KanMX).*

**MD860-1,2,3:** Derivative of MD835 with deletion of *BUB1* and containing the plasmid p*MEC1-BUB1-LYS2*. The deletion of BUB1 was created using the same method as described for MD813. *MAT*α *(hta1-htb1)∆::LEU2 (hta2-htb2)∆::TRP1 leu2 his3 ura3 lys2-128Δ trp1 can1-100* pJD150-Kan *(HTA1-HTB1; KanMX)* p*MEC1-BUB1-LYS2 bub1Δ::Hyg).*

**MD827-1,2:** Transformation of MD761-5-16A with the *sml1∆::loxP-Hyg-loxP* deletion. The transformation was performed with the same 1.8 kb PCR fragment used to construct MD825. *MAT*α *(hta1-htb1)∆::LEU2 (hta2-htb2)∆::TRP1 leu2 his3 ura3 lys2-128Δ trp1 can1-100 pAB6 (HTA1-HTB1; URA3) sml1∆::loxP-Hyg-loxP.*

**MD828-1,2:** Derivative of MD827-1,2 that was transformed with pJD150-Kan plasmid and lost pAB6 plasmid. *MAT*α *(hta1-htb1)∆::LEU2 (hta2-htb2)∆::TRP1 leu2 his3 ura3 lys2-128Δ trp1 can1-100 sml1∆::loxP-Hyg-loxP* pJD150-Kan *(HTA1-HTB1; KanMX).*

**MD870-1,2:** Derivative of MD828-1 with *tel1Δ::loxP-Nat-loxP*. The PCR fragment used to delete *TEL1* has been previously described. *MAT*α *(hta1-htb1)∆::LEU2 (hta2-htb2)∆::TRP1 leu2 his3 ura3 lys2-128Δ trp1 can1-100 sml1∆::loxP-Hyg-loxP* pJD150-Kan *(HTA1-HTB1; KanMX) tel1Δ::loxP-Nat-loxP.*

**MD870-1,2 + pSH47-LYS2(Cre):** Transformant of MD870 with plasmid carrying Cre gene (used to delete *Nat* and *Hyg* markers, retaining the *tel1* and *sml1* mutations). The pSH47-LYS2(Cre) plasmid has been previously described. *MAT*α *(hta1-htb1)∆::LEU2 (hta2-htb2)∆::TRP1 leu2 his3 ura3 lys2-128Δ trp1 can1-100 sml1∆::loxP* pJD150-Kan *(HTA1-HTB1; KanMX) tel1Δ::loxP* pSH47-LYS2(Cre)*.*

**MD872-1,2:** Derivative of MD870 + pSH47-LYS2 (Cre) selected for loss of pSH47-LYS2(Cre) plasmid. *MAT*α *(hta1-htb1)∆::LEU2 (hta2-htb2)∆::TRP1 leu2 his3 ura3 lys2-128Δ trp1 can1-100 sml1∆:: loxP* pJD150-Kan *(HTA1-HTB1; KanMX) tel1Δ::loxP.*

**MD874-1,2:** Derivative of MD872-1,2 containing pMEC1-BUB1-LYS2 and *mec1Δ::Nat.* The plasmid pMEC1-BUB1-LYS2 and the PCR fragment used to delete *MEC1* are described above. *MAT*α *(hta1-htb1)∆::LEU2 (hta2-htb2)∆::TRP1 leu2 his3 ura3 lys2-128Δ trp1 can1-100 sml1∆::loxP* pJD150-Kan *(HTA1-HTB1; KanMX) tel1Δ::loxP mec1Δ::Nat* pMEC1-BUB1-LYS2*.*

**MD878-1,2,3:** Derivative of MD874-1 with deletion of *BUB1*. The PCR fragment used to make the deletion was described in the construction of MD813. *MAT*α *(hta1-htb1)∆::LEU2 (hta2-htb2)∆::TRP1 leu2 his3 ura3 lys2-128Δ trp1 can1-100 sml1∆::loxP* pJD150-Kan *(HTA1-HTB1; KanMX) tel1Δ::loxP mec1Δ::Nat* pMEC1-BUB1-LYS2 *bub1Δ::Hyg.*

**RCY278-5A:** *MATα leu2-3,112 his3-11,15 ura3-1 ade2-1 trp1-1 can1-100 RAD5.* Spore derived from RCY278 (Craven and Petes, 2001). Isogenic with W303-1A (Thomas and Rothstein, 1989).

**Construction and genotypes of diploid derivatives used in the study**

**MD761-5:** *MAT***a***/MAT*α *(hta1-htb1)∆::LEU2/(hta1-htb1)∆::LEU2 (hta2-htb2)∆::TRP1/(hta2-htb2)∆::TRP1 ura3-52/ura3 leu2∆1/leu2 lys2-128∆/ lys2-128∆ his3∆200/his3 trp1∆63/trp1 CAN1/can1-100 pAB6 (HTA1-HTB1; URA3).* Cross of FY406 and RCY278-5A.

**MD801:** *MAT***a/***MAT*α *(hta1-htb1)∆::LEU2/hta1-htb1)∆::LEU2 (hta2-htb2)∆::TRP1/ (hta2-htb2)∆::TRP1 ura3-52/ura3 leu2∆/leu2 lys2-128∆/ lys2-128Δ his3∆200/his3 trp1∆63/trp1 CAN1/can1-100 pAB6 (HTA1-HTB1; URA3)* pJD150-HIS3 (*HIS3*-containing centromeric plasmid with *HTA1-HTB1)*. Cross of FY406-pJD150-HIS3 and MD761-5-16A.

**MD802:** Derivative of MD801 lacking pAB6 plasmid. *MAT***a/***MAT*α *(hta1-htb1)∆::LEU2/hta1-htb1)∆::LEU2 (hta2-htb2)∆::TRP1/ (hta2-htb2)∆::TRP1 ura3-52/ura3 leu2∆/leu2 lys2-128∆/ lys2-128Δ his3∆200/his3 trp1∆63/trp1 CAN1/can1-100* pJD150-HIS3 (*HIS3*-containing centromeric plasmid with *HTA1-HTB1)*.

**SGK178/SGK179:** Transformation of MD802 with PCR fragment containing *URA3* gene to insert *URA3* onto chromosome I; this PCR fragment is described in the construction of SGK334/SGK336. *MAT***a/***MAT*α *(hta1-htb1)∆::LEU2/hta1-htb1)∆::LEU2 (hta2-htb2)∆::TRP1/ (hta2-htb2)∆::TRP1 ura3-52/ura3 leu2∆/leu2 lys2-128∆/ lys2-128Δ his3∆200/his3 trp1∆63/trp1 CEN1::URA3/CEN1 CAN1/can1-100* pJD150-HIS3 (*HIS3*-containing centromeric plasmid with *HTA1-HTB1)*.

**MD821:** Derivative of SGK179 resulting from loss of plasmid pJD150-HIS3 (*HIS3*-containing centromeric plasmid with *HTA1-HTB1)* and transformation with pJD190 (*HIS3*-containing centromeric plasmid with *hta1-S122A-HTB1)*. *MAT***a/***MAT*α *(hta1-htb1)∆::LEU2/hta1-htb1)∆::LEU2 (hta2-htb2)∆::TRP1/ (hta2-htb2)∆::TRP1 ura3-52/ura3 leu2∆/leu2 lys2-128∆/ lys2-128Δ his3∆200/his3 trp1∆63/trp1 CEN1::URA3/CEN1 CAN1/can1-100* pJD190 (*HIS3*-containing centromeric plasmid with *hta1-S122A-HTB1)*.

**MD805:** *MAT***a/***MAT*α *(hta1-htb1)∆::LEU2/hta1-htb1)∆::LEU2 (hta2-htb2)∆::TRP1/ (hta2-htb2)∆::TRP1 ura3-52/ura3 leu2∆/leu2 lys2-128∆/ lys2-128Δ his3∆200/his3 trp1∆63/trp1 CAN1/can1-100 pAB6 (HTA1-HTB1; URA3)* pJD151 (*HIS3*-containing centromeric plasmid with *hta1-S129A-HTB1)*. Cross of FY406-pJD151 and MD761-5-16A.

**MD806:** Derivative of MD805 lacking pAB6 plasmid. *MAT***a/***MAT*α *(hta1-htb1)∆::LEU2/hta1-htb1)∆::LEU2 (hta2-htb2)∆::TRP1/ (hta2-htb2)∆::TRP1 ura3-52/ura3 leu2∆/leu2 lys2-128∆/ lys2-128Δ his3∆200/his3 trp1∆63/trp1 CAN1/can1-100)* pJD151 (*HIS3*-containing centromeric plasmid with *hta1-S129A-HTB1)*.

**MD807:** *MAT***a/***MAT*α *(hta1-htb1)∆::LEU2/hta1-htb1)∆::LEU2 (hta2-htb2)∆::TRP1/ (hta2-htb2)∆::TRP1 ura3-52/ura3 leu2∆/leu2 lys2-128∆/ lys2-128Δ his3∆200/his3 trp1∆63/trp1 CAN1/can1-100 pAB6 (HTA1-HTB1; URA3)* pJD190 (*HIS3*-containing centromeric plasmid with *hta1-S122A-HTB1)*. Cross of FY406-pJD190 and MD761-5-16A.

**MD808:** Derivative of MD807 that lacks pAB6 plasmid. *MAT***a/***MAT*α *(hta1-htb1)∆::LEU2/hta1-htb1)∆::LEU2 (hta2-htb2)∆::TRP1/ (hta2-htb2)∆::TRP1 ura3-52/ura3 leu2∆/leu2 lys2-128∆/ lys2-128Δ his3∆200/his3 trp1∆63/trp1 CAN1/can1-100* pJD190 (*HIS3*-containing centromeric plasmid with *hta1-S122A-HTB1)*.

**SGK177:** Derivative of MD808 that was transformed with PCR fragment to insert the *URA3* gene onto one of the chromosome I homologs; this PCR fragment is described in the construction of SGK334/SGK336. *MAT***a/***MAT*α *(hta1-htb1)∆::LEU2/hta1-htb1)∆::LEU2 (hta2-htb2)∆::TRP1/ (hta2-htb2)∆::TRP1 ura3-52/ura3 leu2∆/leu2 lys2-128∆/ lys2-128Δ his3∆200/his3 CEN1::URA3/CEN1 trp1∆63/trp1 CAN1/can1-100* pJD190 (*HIS3*-containing centromeric plasmid with *hta1-S122A-HTB1)*.

**MD809:** *MAT***a/***MAT*α *(hta1-htb1)∆::LEU2/hta1-htb1)∆::LEU2 (hta2-htb2)∆::TRP1/ (hta2-htb2)∆::TRP1 ura3-52/ura3 leu2∆/leu2 lys2-128∆/ lys2-128Δ his3∆200/his3 trp1∆63/trp1 CAN1/can1-100* pAB6 *(HTA1-HTB1; URA3)* pJD197 (*HIS3*-containing centromeric plasmid with *hta1-S122A, S128A-HTB1)*. Cross of FY406-pJD197 and MD761-5-16A.

**MD810:** Derivative of MD809 that lacks pAB6 plasmid. *MAT***a/***MAT*α *(hta1-htb1)∆::LEU2/hta1-htb1)∆::LEU2 (hta2-htb2)∆::TRP1/ (hta2-htb2)∆::TRP1 ura3-52/ura3 leu2∆1/leu2 lys2-128∆/ lys2-128Δ his3∆200/his3 trp1∆63/trp1 CAN1/can1-100* pJD197 (*HIS3*-containing centromeric plasmid with *hta1-S122A, S128A-HTB1)*.

**MD814:** Cross of MD812-1 and MD813-1. *MAT***a/***MAT*α *HTA1 HTA2 /hta1-htb1)∆::LEU2 HTA2 HTB2/ (hta2-htb2)∆::TRP1 ura3-52/ura3 leu2∆1/leu2 lys2-128∆/ lys2-128Δ his3∆200/his3 trp1∆63/trp1 CAN1/can1-100* pAB6*(HTA1-HTB1; URA3) bub1Δ::Hyg/bub1Δ::Hyg.*

**MD815:** Cross of MD812-2 and MD813-2. *MAT***a/***MAT*α *HTA1 HTA2 /hta1-htb1)∆::LEU2 HTA2 HTB2/ (hta2-htb2)∆::TRP1 ura3-52/ura3 leu2∆1/leu2 lys2-128∆/ lys2-128Δ his3∆200/his3 trp1∆63/trp1 CAN1/can1-100* pAB6*(HTA1-HTB1; URA3) bub1Δ::Hyg/bub1Δ::Hyg.*

**MD816:** Derivative of MD814 screened for loss of the pAB6 plasmid. *MAT***a/***MAT*α *HTA1 HTA2 /hta1-htb1)∆::LEU2 HTA2 HTB2/ (hta2-htb2)∆::TRP1 ura3-52/ura3 leu2∆1/leu2 lys2-128∆/ lys2-128Δ his3∆200/his3 trp1∆63/trp1 CAN1/can1-100 bub1Δ::Hyg/bub1Δ::Hyg.*

**MD817:** Loss of pAB6 from MD815. *MAT***a/***MAT*α *HTA1 HTA2 /hta1-htb1)∆::LEU2 HTA2 HTB2/ (hta2-htb2)∆::TRP1 ura3-52/ura3 leu2∆1/leu2 lys2-128∆/ lys2-128Δ his3∆200/his3 trp1∆63/trp1 CAN1/can1-100 bub1Δ::Hyg/bub1Δ::Hyg.*

**MD836-1.** Cross of MD835-1 and MD833-1. *MAT***a/***MAT*α *(hta1-htb1)∆::LEU2/hta1-htb1)∆::LEU2 (hta2-htb2)∆::TRP1/ (hta2-htb2)∆::TRP1 ura3-52/ura3 leu2∆1/leu2 lys2-128∆/ lys2-128Δ his3∆200/his3 trp1∆63/trp1 CAN1/can1-100 CEN1::URA3/CEN1* pJD150-Kan *(HTA1-HTB1; KanMX).*

**MD913.** Derived from MD836-1 by screening for loss of pJD150-Kan and selecting for transformation with pJD151-HIS3. *MAT***a/***MAT*α *(hta1-htb1)∆::LEU2/hta1-htb1)∆::LEU2 (hta2-htb2)∆::TRP1/ (hta2-htb2)∆::TRP1 ura3-52/ura3 leu2∆1/leu2 lys2-128∆/ lys2-128Δ his3∆200/his3 trp1∆63/trp1 CAN1/can1-100 CEN1::URA3/CEN1* pJD151-HIS3 *(hta1-S129A-HTB1; HIS3).*

**MD914.** Derived from MD836-1 by screening for loss of pJD150-Kan and selecting for transformation with pJD197-HIS3 (*hta1-S122A hta1-S129A*-HIS3). *MAT***a/***MAT*α *(hta1-htb1)∆::LEU2/hta1-htb1)∆::LEU2 (hta2-htb2)∆::TRP1/ (hta2-htb2)∆::TRP1 ura3-52/ura3 leu2∆1/leu2 lys2-128∆/ lys2-128Δ his3∆200/his3 trp1∆63/trp1 CAN1/can1-100 CEN1::URA3/CEN1* pJD197-HIS3 (*hta1-S122A hta1-S129A*-HIS3).

**MD838-1.** Transformation of MD836-1 with pJD150-HIS3. *MAT***a/***MAT*α *(hta1-htb1)∆::LEU2/hta1-htb1)∆::LEU2 (hta2-htb2)∆::TRP1/ (hta2-htb2)∆::TRP1 ura3-52/ura3 leu2∆1/leu2 lys2-128∆/ lys2-128Δ his3∆200/his3 trp1∆63/trp1 CAN1/can1-100 CEN1::URA3/CEN1* pJD150-Kan *(HTA1-HTB1; KanMX)* pJD150-HIS3 (*HTA1-HTB1; HIS3).*

**MD842-1,2.** Loss of pJD150-Kan from MD838-1. *MAT***a/***MAT*α *(hta1-htb1)∆::LEU2/hta1-htb1)∆::LEU2 (hta2-htb2)∆::TRP1/ (hta2-htb2)∆::TRP1 ura3-52/ura3 leu2∆1/leu2 lys2-128∆/ lys2-128Δ his3∆200/his3 trp1∆63/trp1 CAN1/can1-100 CEN1::URA3/CEN1* pJD150-HIS3 (*HTA1-HTB1; HIS3).*

**MD840-1.** Transformation of MD836-1 with pJD190. *MAT***a/***MAT*α *(hta1-htb1)∆::LEU2/hta1-htb1)∆::LEU2 (hta2-htb2)∆::TRP1/ (hta2-htb2)∆::TRP1 ura3-52/ura3 leu2∆1/leu2 lys2-128∆/ lys2-128Δ his3∆200/his3 trp1∆63/trp1 CAN1/can1-100 CEN1::URA3/CEN1* pJD150-Kan *(HTA1-HTB1; KanMX)* pJD190 *(hta1-S122A-HTB1; HIS3).*

**MD844-1.** Loss of pJD150-Kan from MD840-1. *MAT***a/***MAT*α *(hta1-htb1)∆::LEU2/hta1-htb1)∆::LEU2 (hta2-htb2)∆::TRP1/ (hta2-htb2)∆::TRP1 ura3-52/ura3 leu2∆1/leu2 lys2-128∆/ lys2-128Δ his3∆200/his3 trp1∆63/trp1 CAN1/can1-100 CEN1::URA3/CEN1* pJD190 *(hta1-S122A-HTB1; HIS3).*

**MD837-1.** Cross of MD835-1 and MD834-1. *MAT***a/***MAT*α *(hta1-htb1)∆::LEU2/hta1-htb1)∆::LEU2 (hta2-htb2)∆::TRP1/ (hta2-htb2)∆::TRP1 ura3-52/ura3 leu2∆1/leu2 lys2-128∆/ lys2-128Δ his3∆200/his3 trp1∆63/trp1 CAN1/can1-100 CEN1::URA3/CEN1* pJD150-Kan (*HTA1-HTB1; KanMX).*

**MD841-1.** Transformation of MD837-1 with pJD190 plasmid. *MAT***a/***MAT*α *(hta1-htb1)∆::LEU2/hta1-htb1)∆::LEU2 (hta2-htb2)∆::TRP1/ (hta2-htb2)∆::TRP1 ura3-52/ura3 leu2∆1/leu2 lys2-128∆/ lys2-128Δ his3∆200/his3 trp1∆63/trp1 CAN1/can1-100 CEN1::URA3/CEN1* pJD150-Kan (*HTA1-HTB1; KanMX)* pJD190 *(hta1-S122A-HTB1; HIS3)*.

**MD845-1.** Loss of pJD150-Kan plasmid from MD841-1. *MAT***a/***MAT*α *(hta1-htb1)∆::LEU2/hta1-htb1)∆::LEU2 (hta2-htb2)∆::TRP1/ (hta2-htb2)∆::TRP1 ura3-52/ura3 leu2∆1/leu2 lys2-128∆/ lys2-128Δ his3∆200/his3 trp1∆63/trp1 CAN1/can1-100 CEN1::URA3/CEN1* pJD190 *(hta1-S122A-HTB1; HIS3)*.

**MD831.** Cross of haploids MD828-1 and MD829-1. *MAT***a/***MAT*α *(hta1-htb1)∆::LEU2/hta1-htb1)∆::LEU2 (hta2-htb2)∆::TRP1/ (hta2-htb2)∆::TRP1 ura3-52/ura3 leu2∆1/leu2 lys2-128∆/ lys2-128Δ his3∆200/his3 trp1∆63/trp1 CAN1/can1-100 CEN1::URA3/CEN1 sml1∆::Hyg/sml1∆::Hyg* pJD150-Kan *(HTA1-HTB1; KanMX).*

**MD850-1:** Derivative of MD831 screened for loss of pJD150-Kan and transformation with pJD150-HIS3. *MAT*a/*MATα* *(hta1-htb1)∆::LEU2/hta1-htb1)∆::LEU2 (hta2-htb2)∆::TRP1/ (hta2-htb2)∆::TRP1 ura3-52/ura3 leu2∆1/leu2 lys2-128∆/ lys2-128Δ his3∆200/his3 trp1∆63/trp1 CAN1/can1-100 CEN1::URA3/CEN1 sml1∆::Hyg/sml1∆::Hyg* pJD150-HIS3 [*HTA1-HTB1; HIS3].*

**MD851-1.** Derivative of MD831 that lost pJD150-Kan *(HTA1-HTB1; KanMX)* gained pJD190. *MAT***a/***MAT*α *(hta1-htb1)∆::LEU2/hta1-htb1)∆::LEU2 (hta2-htb2)∆::TRP1/ (hta2-htb2)∆::TRP1 ura3-52/ura3 leu2∆1/leu2 lys2-128∆/ lys2-128Δ his3∆200/his3 trp1∆63/trp1 CAN1/can1-100 CEN1::URA3/CEN1 sml1∆::Hyg/sml1∆::Hyg* pJD190*(hta1-S122A-HTB1; HIS3).*

**MD852-1,2:** Isogenic with MD850. Derivative of MD832 screened for loss of pJD150-Kan and gain of pJD150-HIS3. MATa/*MATα* *(hta1-htb1)∆::LEU2/hta1-htb1)∆::LEU2 (hta2-htb2)∆::TRP1/ (hta2-htb2)∆::TRP1 ura3-52/ura3 leu2∆1/leu2 lys2-128∆/ lys2-128Δ his3∆200/his3 trp1∆63/trp1 CAN1/can1-100 CEN1::URA3/CEN1 sml1∆::Hyg/sml1∆::Hyg* pJD150-HIS3 [*HTA1-HTB1; HIS3].*

**MD853-1,2:** Screen for loss of pJD150-kan and select for transformation with pJD190 in strain MD832. MATa/*MATα* *(hta1-htb1)∆::LEU2/hta1-htb1)∆::LEU2 (hta2-htb2)∆::TRP1/(hta2-htb2)∆::TRP1 ura3-52/ura3 leu2∆1/leu2 lys2-128∆/ lys2-128Δ his3∆200/his3 trp1∆63/trp1 CAN1/can1-100 CEN1::URA3/CEN1 sml1∆::Hyg/sml1∆::Hyg* pJD190*(hta1-S122A-HTB1; HIS3)*. Should be isogenic with MD851-1.

**MD832:** Cross of MD828-1 x MD830-1: *MAT***a/***MAT*α *(hta1-htb1)∆::LEU2/hta1-htb1)∆::LEU2 (hta2-htb2)∆::TRP1/(hta2-htb2)∆::TRP1 ura3-52/ura3 leu2∆1/leu2 lys2-128∆/ lys2-128Δ his3∆200/his3 trp1∆63/trp1 CAN1/can1-100 CEN1::URA3/CEN1 sml1∆::Hyg/sml1∆::Hyg* pJD150-Kan *(HTA1-HTB1; KanMX).* This diploid should be isogenic with MD831.

**MD861-1,2** (Cross of MD859-1 x MD860-3): MATa/*MATα* *(hta1-htb1)∆::LEU2/hta1-htb1)∆::LEU2 (hta2-htb2)∆::TRP1/(hta2-htb2)∆::TRP1 ura3-52/ura3 leu2∆1/leu2 lys2-128∆/ lys2-128Δ his3∆200/his3 trp1∆63/trp1 CAN1/can1-100 CEN1::URA3/CEN1 bub1Δ::Hyg/bub1Δ::Hyg* p*MEC1-BUB1-LYS2* pJD150-Kan *(HTA1-HTB1; KanMX).* Same genotype as MD862-1,2.

**SGK659.** Transform MD861-1 with pJD190*(hta1-S122A-HTB1; HIS3)*, and screen for loss pf pJD150-Kan *(HTA1-HTB1; KanMX)* and p*MEC1-BUB1-LYS2*. *MAT***a**/*MATα* *(hta1-htb1)∆::LEU2/hta1-htb1)∆::LEU2 (hta2-htb2)∆::TRP1/(hta2-htb2)∆::TRP1 ura3-52/ura3 leu2∆1/leu2 lys2-128∆/ lys2-128Δ his3∆200/his3 trp1∆63/trp1 CAN1/can1-100 CEN1::URA3/CEN1 bub1Δ::Hyg/bub1Δ::Hyg* pJD190 *(hta1-S122A-HTB1; HIS3).* Same genotype as SGK663.

**MD862-1,2** (MD858-2 x MD860-2): MATa/*MATα* *(hta1-htb1)∆::LEU2/hta1-htb1)∆::LEU2 (hta2-htb2)∆::TRP1/(hta2-htb2)∆::TRP1 ura3-52/ura3 leu2∆1/leu2 lys2-128∆/ lys2-128Δ his3∆200/his3 trp1∆63/trp1 CAN1/can1-100 CEN1::URA3/CEN1 bub1Δ::Hyg/bub1Δ::Hyg* p*MEC1-BUB1-LYS2* pJD150-Kan *(HTA1-HTB1; KanMX).* Same genotype as MD861-1,2.

**SGK663.** Transform MD862-1 with pJD190*(hta1-S122A-HTB1; HIS3)*, and screen for loss pf pJD150-Kan *(HTA1-HTB1; KanMX)* and p*MEC1-BUB1-LYS2*. *MAT***a**/*MATα* *(hta1-htb1)∆::LEU2/hta1-htb1)∆::LEU2 (hta2-htb2)∆::TRP1/(hta2-htb2)∆::TRP1 ura3-52/ura3 leu2∆1/leu2 lys2-128∆/ lys2-128Δ his3∆200/his3 trp1∆63/trp1 CAN1/can1-100 CEN1::URA3/CEN1 bub1Δ::Hyg/bub1Δ::Hyg* pJD190 *(hta1-S122A-HTB1; HIS3).* Same genotype as SGK659.

**MD865-1,2:** Derivative of MD861 that was transformed with pJD150-HIS3 and lost pJD150-Kan and pMEC1-BUB1-LYS2. MATa/*MATα* *(hta1-htb1)∆::LEU2/hta1-htb1)∆::LEU2 (hta2-htb2)∆::TRP1/(hta2-htb2)∆::TRP1 ura3-52/ura3 leu2∆1/leu2 lys2-128∆/ lys2-128Δ his3∆200/his3 trp1∆63/trp1 CAN1/can1-100 CEN1::URA3/CEN1* pJD150-HIS3 [*HTA1-HTB1; HIS3]*  *bub1Δ::Hyg/bub1Δ::Hyg.* Same genotype as MD868-1,2.

**MD868-1,2:** Derivative of MD862 transformed with pJD150-HIS3 and lost pJD150-Kan and pMEC1-BUB1-LYS2. MATa/*MATα* *(hta1-htb1)∆::LEU2/hta1-htb1)∆::LEU2 (hta2-htb2)∆::TRP1/(hta2-htb2)∆::TRP1 ura3-52/ura3 leu2∆1/leu2 lys2-128∆/ lys2-128Δ his3∆200/his3 trp1∆63/trp1 CAN1/can1-100 CEN1::URA3/CEN1* pJD150-HIS3 [*HTA1-HTB1; HIS3]*  *bub1Δ::Hyg/bub1Δ::Hyg.* Same genotype as MD865-1,2.

**MD876-1,2** (Cross MD875-1xMD874-1): MATa/*MATα* *(hta1-htb1)∆::LEU2/hta1-htb1)∆::LEU2 (hta2-htb2)∆::TRP1/(hta2-htb2)∆::TRP1 ura3-52/ura3 leu2∆1/leu2 lys2-128∆/ lys2-128Δ his3∆200/his3 trp1∆63/trp1 CEN1::URA3/CEN1 sml1Δ::loxP/sml1Δ::loxP* pJD150-Kan *[HTA1-HTB1; KanMX) tel1Δ::loxP/tel1Δ::loxP* p*MEC1-BUB1-LYS2 mec1Δ::Nat/mec1Δ::Nat*

**MD877-1,2** (Cross of MD875-1xMD874-1)**:** MATa/*MATα* *(hta1-htb1)∆::LEU2/hta1-htb1)∆::LEU2 (hta2-htb2)∆::TRP1/(hta2-htb2)∆::TRP1 ura3-52/ura3 leu2∆1/leu2 lys2-128∆/ lys2-128Δ his3∆200/his3 trp1∆63/trp1 CAN1/can1-100 CEN1::URA3/CEN1 sml1Δ::loxP/sml1Δ::loxP* pJD150-Kan *[HTA1-HTB1; KanMX) tel1Δ::loxP/tel1Δ::loxP* p*MEC1-BUB1-LYS2 mec1Δ::Nat/mec1Δ::Nat*

**MD915:** Selection of a derivative of MD877 that lacks the pJD50-Kan and p*MEC1-BUB2-LYS2* plasmids and contains the pJD150-HIS3 plasmid. MATa/*MATα* *(hta1-htb1)∆::LEU2/hta1-htb1)∆::LEU2 (hta2-htb2)∆::TRP1/(hta2-htb2)∆::TRP1 ura3-52/ura3 leu2∆1/leu2 lys2-128∆/ lys2-128Δ his3∆200/his3 trp1∆63/trp1 CAN1/can1-100 CEN1::URA3/CEN1 sml1Δ::Hyg/sml1Δ::Hyg* pJD150-HIS3 *[HTA1-HTB1; HIS3) tel1Δ::loxP/tel1Δ::loxP mec1Δ::Nat/mec1Δ::Nat*

**MD917:** Derivative of MD876 that lacks the pJD50-Kan and p*MEC1-BUB2-LYS2* plasmids and contains the pJD190-HIS3 plasmid. MATa/*MATα* *(hta1-htb1)∆::LEU2/hta1-htb1)∆::LEU2 (hta2-htb2)∆::TRP1/(hta2-htb2)∆::TRP1 ura3-52/ura3 leu2∆1/leu2 lys2-128∆/ lys2-128Δ his3∆200/his3 trp1∆63/trp1 CAN1/can1-100 CEN1::URA3/CEN1 sml1Δ::Hyg/sml1Δ::Hyg* *tel1Δ::loxP/tel1Δ::loxP mec1Δ::Nat/mec1Δ::Nat* pJD190*(hta1-S122A-HTB1; HIS3)*.

**MD918:** Derivative of MD877 that lacks the pJD50-Kan and p*MEC1-BUB2-LYS2* plasmids and contains the pJD190-HIS3 plasmid. Isogenic with MD917. MATa/*MATα* *(hta1-htb1)∆::LEU2/hta1-htb1)∆::LEU2 (hta2-htb2)∆::TRP1/(hta2-htb2)∆::TRP1 ura3-52/ura3 leu2∆1/leu2 lys2-128∆/ lys2-128Δ his3∆200/his3 trp1∆63/trp1 CAN1/can1-100 CEN1::URA3/CEN1 sml1Δ::Hyg/sml1Δ::Hyg* *tel1Δ::loxP/tel1Δ::loxP mec1Δ::Nat/mec1Δ::Nat* pJD190*(hta1-S122A-HTB1; HIS3)*.

**MD880-1** (Cross of MD879-2 x MD878-3): MATa/*MATα* *(hta1-htb1)∆::LEU2/hta1-htb1)∆::LEU2 (hta2-htb2)∆::TRP1/(hta2-htb2)∆::TRP1 ura3-52/ura3 leu2∆1/leu2 lys2-128∆/ lys2-128Δ his3∆200/his3 trp1∆63/trp1 CAN1/can1-100 CEN1::URA3/CEN1 sml1Δ::loxP/sml1Δ::loxP* *tel1Δ::loxP/tel1Δ::loxP mec1Δ::Nat/mec1Δ::Nat* pJD150-Kan *[HTA1-HTB1; KanMX)* p*MEC1-BUB1-LYS2 bub1Δ::Hyg/bub1Δ::Hyg*

**MD881-1** (Cross of MD879-1 x MD878-1): MATa/*MATα* *(hta1-htb1)∆::LEU2/hta1-htb1)∆::LEU2 (hta2-htb2)∆::TRP1/(hta2-htb2)∆::TRP1 ura3-52/ura3 leu2∆1/leu2 lys2-128∆/ lys2-128Δ his3∆200/his3 trp1∆63/trp1 CAN1/can1-100 CEN1::URA3/CEN1 sml1Δ::loxP/sml1Δ::loxP* *tel1Δ::loxP/tel1Δ::loxP mec1Δ::Nat/mec1Δ::Nat* pJD150-Kan *[HTA1-HTB1; KanMX)* p*MEC1-BUB1-LYS2 bub1Δ::Hyg/bub1Δ::Hyg*

**MD882-2/MD883-1:** Derived from MD880 by loss of the plasmids pMEC1-BUB1-LYS2 and pJD150-Kan, and transformation with plasmid pJD150-HIS3. MATa/*MATα* *(hta1-htb1)∆::LEU2/hta1-htb1)∆::LEU2 (hta2-htb2)∆::TRP1/(hta2-htb2)∆::TRP1 ura3-52/ura3 leu2∆1/leu2 lys2-128∆/ lys2-128Δ his3∆200/his3 trp1∆63/trp1 CAN1/can1-100 CEN1::URA3/CEN1 sml1Δ::Hyg/sml1Δ::Hyg* *tel1Δ::loxP/tel1Δ::loxP mec1Δ::Nat/mec1Δ::Nat* pJD150-HIS3 *[HTA1-HTB1; HIS3)* *bub1Δ::Hyg/bub1Δ::Hyg*

**MD884-1/MD885-1:** Derived from MD881-1 by loss of the plasmids pMEC1-BUB1-LYS2 and pJD150-Kan, and transformation with plasmid pJD190-HIS3. MATa/*MATα* *(hta1-htb1)∆::LEU2/hta1-htb1)∆::LEU2 (hta2-htb2)∆::TRP1/(hta2-htb2)∆::TRP1 ura3-52/ura3 leu2∆1/leu2 lys2-128∆/ lys2-128Δ his3∆200/his3 trp1∆63/trp1 CAN1/can1-100 CEN1::URA3/CEN1 sml1Δ::Hyg/sml1Δ::Hyg* *tel1Δ::loxP/tel1Δ::loxP mec1Δ::Nat/mec1Δ::Nat* pJD190-HIS3 *[hta1-S122A; HIS3)* *bub1Δ::Hyg/bub1Δ::Hyg.*

**MD1002:** (Cross of MD1000-1 and MD835-1). *MAT***a/***MAT*α *(hta1-htb1)∆::LEU2/hta1-htb1)∆::LEU2 (hta2-htb2)∆::TRP1/ (hta2-htb2)∆::TRP1 ura3-52/ura3 leu2∆1/leu2 lys2-128∆/ lys2-128Δ his3∆200/his3 trp1∆63/trp1 CAN1/can1-100 CEN1::URA3/CEN1 I-9500::Hyg/I-9500* pJD150-Kan *(hta1-S122A-HTB1;KanMX).*

**MD1003:** (Cross of MD1001-1 and MD874-1). *MAT***a/***MAT*α *(hta1-htb1)∆::LEU2/hta1-htb1)∆::LEU2 (hta2-htb2)∆::TRP1/ (hta2-htb2)∆::TRP1 ura3-52/ura3 leu2∆1/leu2 lys2-128∆/ lys2-128Δ his3∆200/his3 trp1∆63/trp1 CAN1/can1-100 sml1Δ::loxP/sml1Δ::loxP* *tel1Δ::loxP/tel1Δ::loxP mec1Δ::Nat/mec1Δ::Nat CEN1::URA3/CEN1 I-9500::Hyg/I-9500* pJD150-Kan *(hta1-S122A-HTB1;KanMX)* p*MEC1-BUB1-LYS2.*

**MD1004:** Derivative of MD1002 that was transformed with pJD190-HIS3 and lost the plasmid pJD150-Kan *(hta1-S122A-HTB1;KanMX).* *MAT***a/***MAT*α *(hta1-htb1)∆::LEU2/hta1-htb1)∆::LEU2 (hta2-htb2)∆::TRP1/ (hta2-htb2)∆::TRP1 ura3-52/ura3 leu2∆1/leu2 lys2-128∆/ lys2-128Δ his3∆200/his3 trp1∆63/trp1 CAN1/can1-100 CEN1::URA3/CEN1 I-9500::Hyg/I-9500* pJD190-HIS3 *(hta1-S122A-HTB1; HIS3).*

**MD1005:** Derivative of MD1003 that lost the pJD150-Kan and p*MEC1-BUB1-LYS2* plasmids and was transformed with the pJD190-HIS3 plasmid. *MAT***a/***MAT*α *(hta1-htb1)∆::LEU2/hta1-htb1)∆::LEU2 (hta2-htb2)∆::TRP1/ (hta2-htb2)∆::TRP1 ura3-52/ura3 leu2∆1/leu2 lys2-128∆/ lys2-128Δ his3∆200/his3 trp1∆63/trp1 CAN1/can1-100 sml1Δ::loxP/sml1Δ::loxP* *tel1Δ::loxP/tel1Δ::loxP mec1Δ::Nat/mec1Δ::Nat CEN1::URA3/CEN1 I-9500::Hyg/I-9500* pJD190-HIS3 *(hta1-S122A-HTB1; HIS3).*

**MD1010:** Derivative of MD1003 that lost the pJD150-Kan and p*MEC1-BUB1-LYS2* plasmids and was transformed with the pJD150-HIS3 plasmid. *MAT***a/***MAT*α *(hta1-htb1)∆::LEU2/hta1-htb1)∆::LEU2 (hta2-htb2)∆::TRP1/ (hta2-htb2)∆::TRP1 ura3-52/ura3 leu2∆1/leu2 lys2-128∆/ lys2-128Δ his3∆200/his3 trp1∆63/trp1 CAN1/can1-100 sml1Δ::loxP/sml1Δ::loxP* *tel1Δ::loxP/tel1Δ::loxP mec1Δ::Nat/mec1Δ::Nat* CEN1*::URA3/CEN1 I-9500::Hyg/I-9500* pJD150-HIS3 *[HTA1-HTB1; HIS3).*

**References**

Hirschhorn JN, Bortvin AL, Ricupero-Hovasse SL, and Winston F (1995). A new class of histone H2A mutations in *Saccharomyces cerevisiae* causes specific transcriptional defects in vivo. Mol. Cell. Biol. 15: 1999-2009.

Goldstein AL, and McCusker JH (1999). Three new dominant drug resistance cassettes for gene disruption in *Saccharomyces cerevisiae*. Yeast 15: 1541-1553.

Harvey AC, Jackson SP, and Downs, JA (2005). *Saccharomyces cerevisiae* Histone H2A Ser122 facilitates DNA repair. Genetics 170: 543-553.

Craven RJ and Petes TD (2001). The *Saccharomyces cerevisiae* suppressor of choline sensitivity (*SCS2*) is a multicopy suppressor of mec1 telomere silencing defects. Genetics 158: 145-154.

Thomas BJ and Rothstein R (1989). Elevated recombination rates in transcriptionally active DNA. Cell 56: 619-630.

Guo X, Hum YF, Lehner K, and Jinks-Robertson S (2017). Regulation of hetDNA length during mitotic double-strand break repair in yeast. Mol. Cell 67: 539-549.

Hirschhorn JN, Bortvin AL, Ricupero-Hovasse SL, and Winston F (1995). A new class of histone H2A mutations in Saccharomyces cerevisiae causes specific transcriptional defects in vivo. Mol. Cell. Biol. 15: 1999-2009.

Goldstein AL, and McCusker JH (1999). Three new dominant drug resistance cassettes for gene disruption in *Saccharomyces cerevisiae*. Yeast 15: 1541-1553.

Harvey AC, Jackson SP, and Downs, JA (2005). *Saccharomyces cerevisiae* Histone H2A Ser122 facilitates DNA repair. Genetics 170: 543-553.

Craven RJ and Petes TD (2001). The *Saccharomyces cerevisiae* suppressor of choline sensitivity (*SCS2*) is a multicopy suppressor of mec1 telomere silencing defects. Genetics 158: 145-154.

Thomas BJ and Rothstein R (1989). Elevated recombination rates in transcriptionally active DNA. Cell 56: 619-630.

Guo X, Hum YF, Lehner K, and Jinks-Robertson S (2017). Regulation of hetDNA length during mitotic double-strand break repair in yeast. Mol. Cell 67: 539-549.
